# Blocking nuclear export of HSPA8 after heat shock stress severely alters cell survival

**DOI:** 10.1038/s41598-018-34887-6

**Published:** 2018-11-14

**Authors:** Fengjuan Wang, Srinivasa Reddy Bonam, Nicolas Schall, Lauriane Kuhn, Philippe Hammann, Olivier Chaloin, Jean-Baptiste Madinier, Jean-Paul Briand, Nicolas Page, Sylviane Muller

**Affiliations:** 1 CNRS-University of Strasbourg, Biotechnology and cell signaling, Illkirch, France/Laboratory of excellence Medalis, Strasbourg, France; 2 CNRS, Proteomics Facilities, Institut de Biologie Moléculaire et Cellulaire, Strasbourg, France; 3 CNRS, Immunology, Immunopathology and Therapeutic Chemistry, Institut de Biologie Moléculaire et Cellulaire, Strasbourg, France; 4 Department of Pathology and Immunology, CMU-University of Geneva, Geneva, Switzerland; 5 University of Strasbourg Institute for Advanced Study, Strasbourg, France

## Abstract

The nuclear translocation of endogenous heat shock cognate protein HSPA8 is a requisite for cell survival during oxidative and heat shock stress. Upon these events, cytoplasmic HSPA8 is thought to concentrate within the nucleus and nucleolus. When the situation returns to normal, HSPA8 is released from its nuclear/nucleolar anchors and redistributes into the cytoplasm. By using different stress conditions and a 21-mer phosphopeptide tool called P140, which binds HSPA8 and hampers its chaperone properties, we deciphered the cellular and molecular effects arising during this vital cytoplasmic-nuclear-cytoplasmic shuttling process. Using the non-metastatic fibroblastoid cell line MRL/N-1 derived from a MRL/MpTn-gld/gld lupus-prone mouse, we discovered that P140 treatment neutralized the egress of HSPA8 from nucleus to cytoplasm in the cell recovery phase. This lack of relocation of HSPA8 into the cytoplasm of heat-shocked MRL/N-1 cells altered the ability of these cells to survive when a second mild oxidative stress mimicking inflammatory conditions was applied. Crosslinking experiments followed by proteomics studies showed that P140 binds regions close to nuclear import and export signal sequences encompassed within the HSPA8 structure. These data are consistent with HSPA8 having a crucial cell protective role against reactive oxygen species (ROS) production by mitochondria during inflammatory conditions.

## Introduction

In contrast to cytoplasmic HSP70s that are generated in response to stress, proteins of the HSPA8/HSC70 family are constitutively expressed. They exhibit pleiotropic properties and are crucial for cell survival^1,2^. In particular, HSPA8 proteins participate to the folding of nascent proteins or refolding of altered proteins, and to their targeting to the ubiquitin/proteasome machinery for degradation. HSPA8 is involved in protein import into organelles or cellular compartments. Under normal conditions, cytoplasmic HSPA8 shuttles between the cytoplasm and the nucleus in an ATP-dependent manner^3^, a property that enables HSPA8 to import different cytoplasmic (client) proteins into the nucleus^4^. To translocate within the nucleus, HSPA8 either interacts with nuclear localization sequence (NLS)-containing proteins or exploits its own NLS. At least two NLS sequences have been identified so far in human HSPA8, both located within the nucleotide binding domain^5–7^. They are present in residues 69-DAKRL-73 in the N-terminus and 246-KRKHKKDISENKRAVRR-262 in the ATPase domain of HSPA8. A nucleolar localization signal (NoLS) sufficient to promote nucleolar targeting in response to heat shock (HS) was identified in residues 225–244 of HSPA8^8^. Within the tertiary structure of the N-terminal HSPA8 ATPase domain (nucleotide-binding domain, NBD), this segment is located within the domain B of lobe II, which encompasses residues 229–306^9,10^. The shuttling capacity of HSPA8 also contributes to the cytosolic export of nuclear proteins, a mechanism that requires energy input^11^. A nuclear export signal (NES) motif has been identified in residues 394–401 of HSPA8 at the very N-terminus of substrate-binding domain^7^. The signaling pathways involved in HSPA8 trafficking in case of stress are not known in all details. While HSPA8 normally shuttles between the cytoplasm (where it is extremely abundant) and the nucleus, in case of heat-induced stress, its nuclear export is transiently inhibited, confining HSPA8 within the nucleus^12^. In certain stress conditions, notably when cells are exposed to HS or oxidative stress produced by hydrogen peroxide (H_2_O_2_), HSPA8 can concentrate in nucleoli. When the physiological situation returns to normal (recovery phase from stress), HSPA8 proteins are released from nuclear and nucleolar anchors and its shuttling to cytoplasm is restored. The duration of this process (in the range of several hours) varies according to the type of cells and stress.

It has been described that confluent or high density cell cultures could have negative impact on nucleocytoplasmic trafficking of HSPA8, thereby preventing its nuclear import^13^. Several pharmacological inhibitors were also found to hamper HSPA8 import or export. Thus, the phosphoinositide 3-kinase inhibitor LY294002 was used to demonstrate (partial) inhibition of nucleolar accumulation of HSPA8 during the recovery phase^8^. Indomethacin and Ibuprofen, which are non-steroidal anti-inflammatory drugs, have been shown to mobilize HSPA8 in the cell nucleus^14^. Having in hands an original peptide, called P140, which was found to readily bind HSPA8 both *in vitro* and *in vivo*, to interact with the NBD of HSPA8 and shown to inhibit some of the protein functions^15–17^, we addressed the question of whether P140 peptide could affect HSPA8 homing into nuclei and nucleoli under stress conditions and recovery, and what the cellular consequences would be. The uppermost interest of investigating this question with P140 originates from the fact that this peptide, a 21-mer fragment of the 70 kDa spliceosomal small nuclear ribonucleoprotein (SNRNP70/U1-70K) protein, is a potent immunomodulator of lupus disease as shown in lupus-prone MRL/lpr mice^18,19^ and autoimmune patients with systemic lupus erythematosus^20^. It is currently evaluated in a phase III-clinical trial (program Lupuzor). Furthermore, knowing that HSPA8 also acts as a chaperone for class II major histocompatibility complex proteins^21–25^, any alteration of its trafficking may potentially affect antigenic peptide presentation to the receptor of T cells, leading to a possible regulation of the downstream immune response.

First, we found that according to the type of injury that was applied to cells (HS or oxidative stress), the cytoplasmic-nuclear shuttling capacities of HSPA8 were differently regulated. Then, using the P140 peptide as an HSPA8 binder, we unexpectedly discovered that in the presence of this peptide, HSPA8 did not egress from the nucleus to the cytoplasm in the cell recovery phase but remained sequestered into the nucleus. We found that one of the serious consequences of this defective relocation of HSPA8 into the cytoplasm was the inability of heat-shocked cells to survive after a second type of aggression, e.g. a mild oxidative stress that virtually mimics a state of inflammation. Crosslinking experiments followed by proteomics studies allowed us to demonstrate that these effects of P140 likely result from its binding to regions close to nuclear import and export signal sequences encompassed within the HSPA8 structure.

## Results

### Nucleocytoplasmic shuttling of endogenous HSPA8 in heat-shocked MRL/N-1 cells

**Figure 1:**
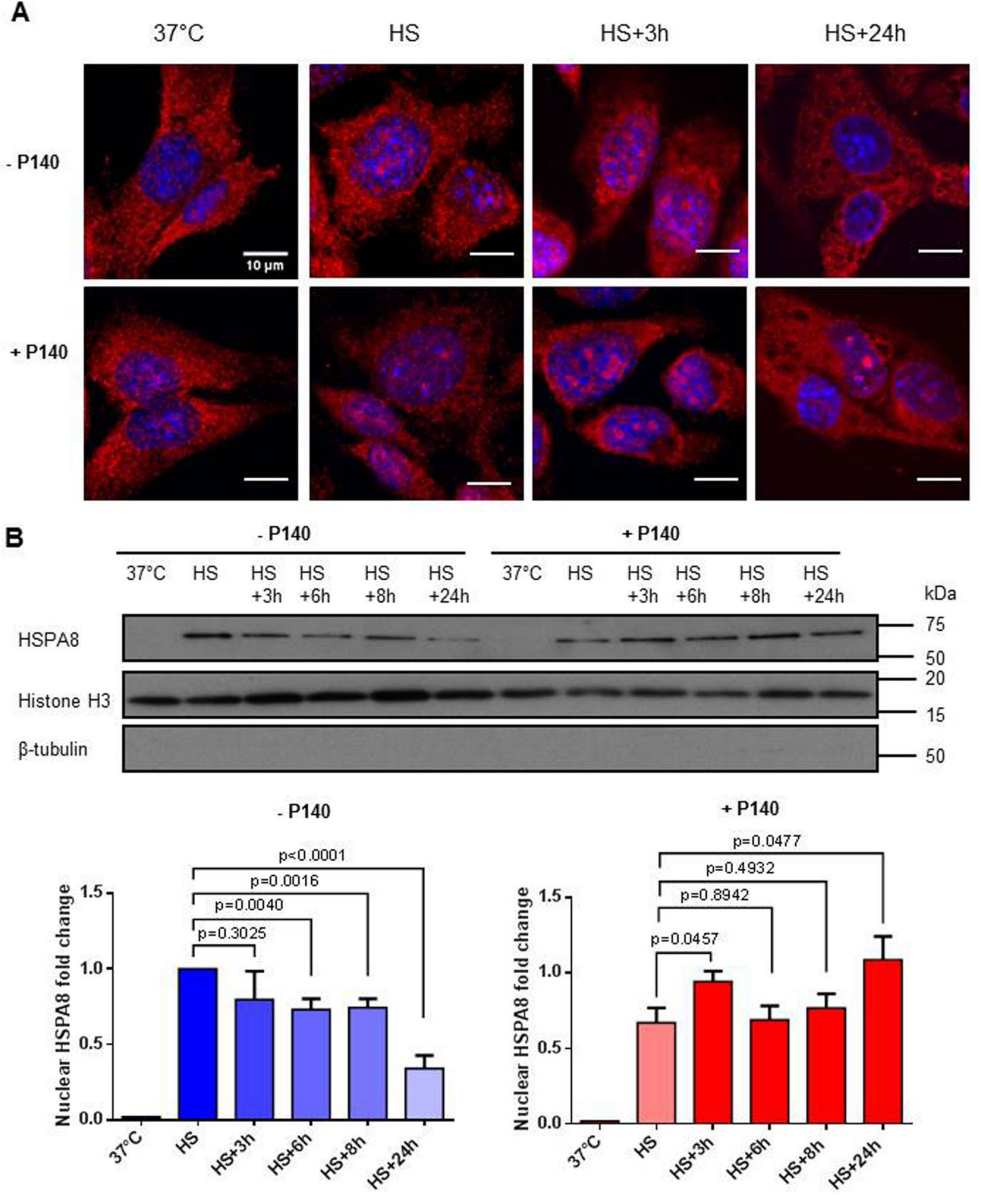
P140 affects the nuclear translocation of HSPA8 upon heat shock. (**A**) Confocal fluorescence images of cellular HSPA8 upon HS and recovery in the presence or absence of P140. MRL/N-1 cells were exposed to HS (30 min; 45 °C), and allowed to recover at 37 °C for indicated time points (3 and 24 h) following HS, in the absence or presence of 10 µM P140. Cells were stained with HSPA8-PE antibody (in red). The nuclei were stained with DAPI (in blue). Transmission images showing the morphology of cells are presented in Fig. S1A. (**B**) Western immunoblotting of nuclear HSPA8 upon HS and recovery in the presence or absence of P140. MRL/N-1 cells were treated and allowed to recover as in (**A**). Cells were collected at the indicated time points of the recovery phase and subjected to cell fractionation to isolate the nuclear and cytoplasmic fractions. Nuclear HSPA8 only was analyzed here. Histone H3 was used as nuclear marker and β-tubulin as cytosolic marker. The relative amount of nuclear HSPA8 was analyzed by densitometry with Image J. The fold change was plotted by normalizing the nuclear HSPA8 amounts by that of the control after HS (value set to 1). Mean values with standard error of the mean (SEM) of five independent experiments are presented. Non-parametric two-way ANOVA was used to evaluate the statistical significance.

While in unstressed conditions most of HSPA8 widely distributes inside the cytoplasm, it has been previously described that upon HS or oxidative stress, HSPA8 translocates into nucleus and transiently concentrates inside nucleolus. During recovery from stress conditions, nuclear HSPA8 relocates to cytoplasm^8^. In preliminary experiments performed by confocal microscopy and western immunoblotting (Fig. 1A,B; Fig. S1), we verified that this process did occur in the non-metastatic fibroblastoid cell line MRL/N-1. This cell line was selected for conducting our studies because it was derived from the spleen of an MRL/MpTn-gld/gld (MRL/gld) mouse^26,27^, a murine model close to lupus-prone MRL/lpr mice in which our previous studies were conducted^15–19^. As MRL/lpr mice, MRL/gld mice are deficient in a functional Fas ligand and spontaneously develop autoimmune diseases involving both lethal glomerulonephritis and systemic arteritis. We effectively confirmed the whole process of HSPA8 nuclear/nucleolar import upon heat stress and cytoplasm relocation throughout the recovery phase in this cell line. Nucleus HSPA8 egress was detectable between 3 and 6 h onwards (Fig. 1B). As anticipated due to its large abundance in the cytoplasm^28,29^, it was not possible to detect any variation of the HSPA8 (and HSP90) content in the cytoplasmic and total fraction (Figs S2 and S3).

### P140 alters the nucleocytoplasmic shuttling of endogenous HSPA8 upon heat shock stress

We then repeated these experiments using MRL/N-1 cells incubated with 10 µM P140 peptide, and observed striking effects of the peptide on HSPA8 trafficking and cell growth. P140 slightly slowed down the entry of endogenous HSPA8 into the nucleus upon HS but more importantly, prolonged its sequestration in the nucleus/nucleoli upon the recovery phase (Fig. 1; Fig. S1A). These observations were confirmed in mouse embryonic fibroblasts (MEF) cells used as a second cell type to reinforce our findings (Fig. S4A). The level of HSPA8 in the cytosolic fraction of MRL/N-1 cells was diminished at 24 h compared to the cells that were not treated with P140 peptide (Fig. S2), the total amount of HSPA8 and HSP90 remained apparently unchanged (Fig. S3). Of note, P140 and ScP140 peptides had no effect on HSPA8 mRNA expression levels (Fig. S3E).

### Nucleocytoplasmic shuttling of endogenous HSPA8 does not occur in MRL/N-1 cells subjected to a mild oxidative stress or to nutrient starvation

It is known that HS is the most efficient treatment to rapidly induce HSPA8 accumulation in nuclei. Depending on cell type and stressor compounds and also if short or prolonged exposure to stressors is applied, different effects can be observed on protein trafficking. A series of tests were therefore performed to examine the subcellular distribution of HSPA8 after an oxidative stress induced by H_2_O_2_. We observed that in contrast to what we found in HS stress conditions, mild oxidative stress induced with 100 µM H_2_O_2_ or with Paraquat (not a ROS by itself but generating oxygen radicals through the redox cycling mechanism), or nutrient starvation did not provoke HSPA8 translocation into the nucleus of MRL/N-1 cells and that P140 treatment had no effect on this process (Fig. S5, in the presence of H_2_O_2_; other data not shown).

### Consequences of the P140-induced HSPA8 nuclear sequestration in heat-shocked cells

We have previously seen that in acute HS stress conditions, P140 peptide induces the sequestration of HSPA8 within the nucleus/nucleolus. Hence we wanted to determine whether this lack of return to normalcy might have any effect on cell behavior.

**Figure 2:**
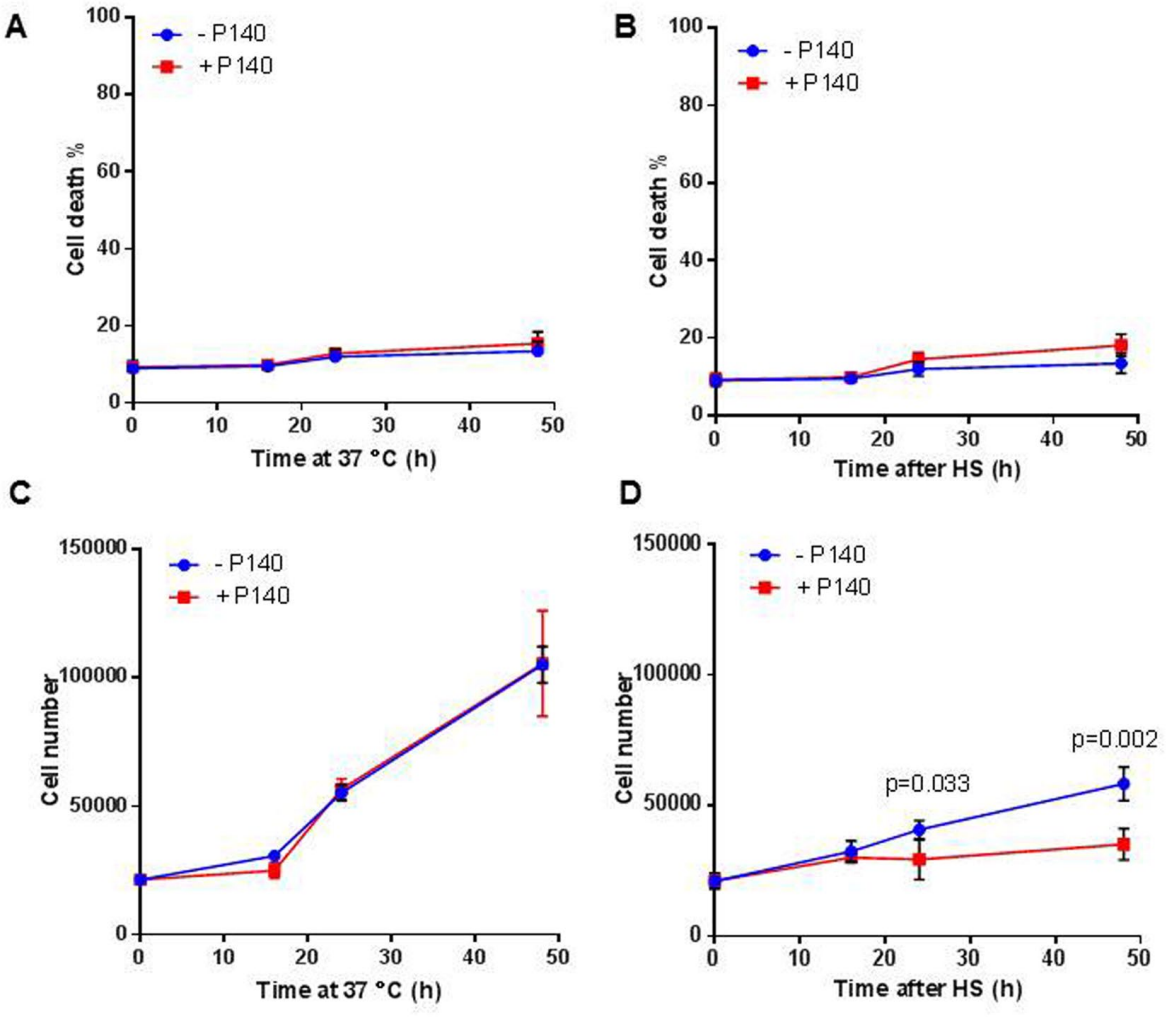
P140 does not affect cell survival but reduces cell growth of heat-shocked MRL/N-1 cells. Cell death percentage (**A,B**) or cell counts (**C,D**) of MRL/N-1 cells that were either kept at 37 °C at all time points (**A,C**) or heat-shocked and left at 37 °C for recovery (**B,D**) in the presence or not of 10 µM P140. Cell death was measured using PI staining and flow cytometry. Cell counts were determined using a LUNA-FL automated cell counter. Mean values ± SD of two independent experiments with triplicate samples are presented. Two-way ANOVA was used to evaluate the statistical significance.

First we examined the viability level of MRL/N-1 cells and their ability to grow. The percentage of propidium iodide positive (PI^+^) cells measured during the post-HS recovery period remained low (<10%) and was not affected by the presence of P140 peptide (Fig. 2A,B). This suggests that the integrity of plasma membrane of heat-shocked cells was preserved, that the basal number of necrotic (and apoptotic) cells was unaffected, and that the influence of P140 on HSPA8 trafficking did not provoke abnormal mortality. In contrast, however, cell counts measured over the recovery time were significantly reduced in the presence of P140 in the cultures (Fig. 2C,D) indicating that P140-induced sequestration of HSPA8 into the nucleus of HS-stressed MRL/N-1 cells altered their proliferation.

It is well established that HSPA8 is involved in the progression of cell cycle during the G1/S transition, notably by regulating the nuclear accumulation of cyclin D1 that is central in G1 to S phase cell cycle transition and *via* its interaction with connexin 43^30–32^. Flow cytometry experiments with MRL/N-1 cells showed that upon HS, P140 had no effect on cell cycle (Fig. S6).

No effect could be detectable either when the intensity of macroautophagy and chaperone-mediated autophagy (CMA) processes was studied (Fig. S7). Autophagy was investigated as HSPA8 plays key functions in this vital process, especially in CMA^2,33,34^, and that abnormal overexpression in B cells from MRL/lpr lupus-prone mice of HSPA8 and lysosomal-associated membrane protein 2A (LAMP2A), another rate-limiting protein in CMA that is responsible for the selective degradation of cytosolic proteins in lysosomes, is down-regulated in P140-treated mice^16,17^. We found no change of the expression of microtubule-associated protein light chain-3 (LC3-II/MAP1LC3-II), sequestosome 1 (SQSTM1/p62; not shown), and LAMP2A in total lysates of HS-stressed MRL/N-1 cells incubated with or without P140.

HSPA8 has also been shown to be essential for regulating cellular signaling and notably participates to Akt signaling pathway in endothelial cells^35^. It was also demonstrated that the nuclear translocation of HSPA8 requires the signaling events through the PI3-kinase → Akt and MEK → ERK1/2 pathways^8^. We therefore studied markers of these two kinase signaling cascades in non-stressed or HS-stressed cells, both in the presence or absence of P140 peptide. The latter showed no effect on the expression of PI3K and MEK-ERK1/2 markers (Fig. S8**)**.

### HSPA8 nuclear sequestration by P140 peptide makes the MRL/N-1 cells more vulnerable to stress conditions

**Figure 3:**
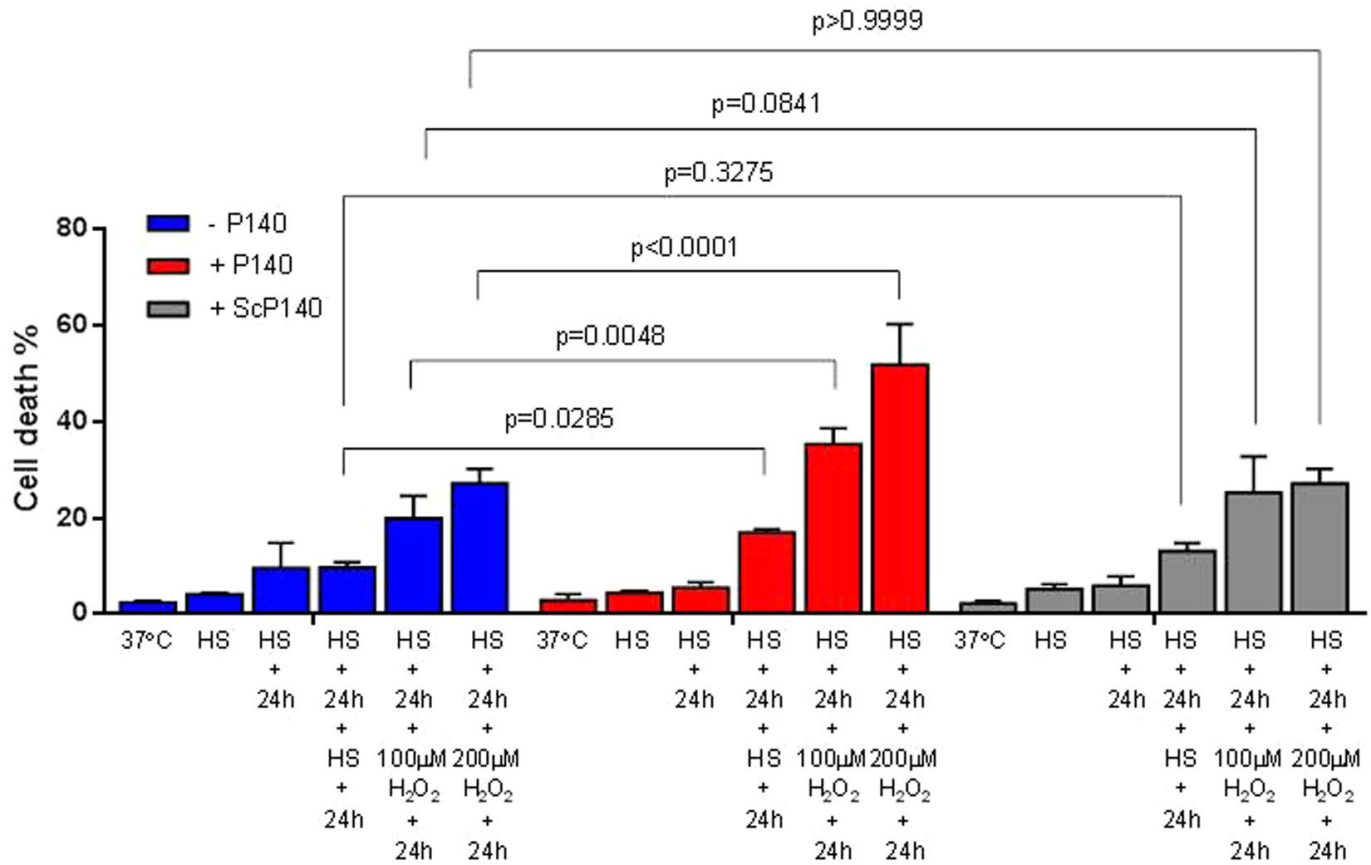
P140 compromises heat-shocked cells upon a second mild oxidative stress. MRL/N-1 cells were heat-shocked and led to recover at 37 °C for 24 h in the presence or absence of P140 and control peptide ScP140. Another shock induced either by HS (30 min at 45 °C) or H_2_O_2_ (for 1 h) was then applied followed by a 24h-recovery time at 37 °C. Cell death was assessed using PI staining and flow cytometry. Mean values ± SD of two independent experiments with triplicates are presented. Two-way ANOVA was used to evaluate the statistical significance.

Because HSPA8 translocation to nuclei/nucleoli is supposed to represent a protecting event against aggression, we subjected heat-shocked MRL/N-1 cells to a second set of stress. In the cultures that were not treated with P140 (Fig. 3, blue bars), we noted that after two sequential HS exposures, separated by 24 h recovery in fresh medium at 37 °C, cultures contained less than 10% PI^+^ cells, which is of the same order of magnitude as what was evaluated after a single HS period. Cell death was slightly raised, although not significantly, in the cultures that were sequentially exposed to an HS and then to an oxidative stress, separated by 24 h recovery. In this case, cell cultures contained about 20–25% PI^+^ cells and this effect was H_2_O_2_ dose-dependent. In the presence of P140 in the cultures (Fig. 3, red bars), the cell death level was impressively increased to reach up to 51.8 ± 4.2% PI^+^ cells, according to the H_2_O_2_ concentration added to the cultures (*P* = 0.0048 and *P* < 0.0001 in the presence of 100 or 200 µM H_2_O_2_, respectively). The thermosensitivity of P140-treated cells (second HS) was also altered but much more modestly (*P* = 0.0285). The control ScP140 peptide had no effect (Fig. 3; gray bars). The same phenomenon was observed in MEF cells (Fig. S4B).

Altogether, our results support the view that by blocking the egress of HSPA8 chaperone from cell nuclei/nucleoli, P140 severely compromises the ability of cells to survive to a subsequent oxidative stress. These findings are of central importance since oxidative stress is one of the key manifestations of inflammation. These results may also be of underlying value to explain, at least in part, how P140 peptide leads to death of activated autoimmune cells^15,16^.

### P140 peptide alters HSPA8 trafficking by blocking the NLS and NES of the chaperone protein

**Figure 4:**
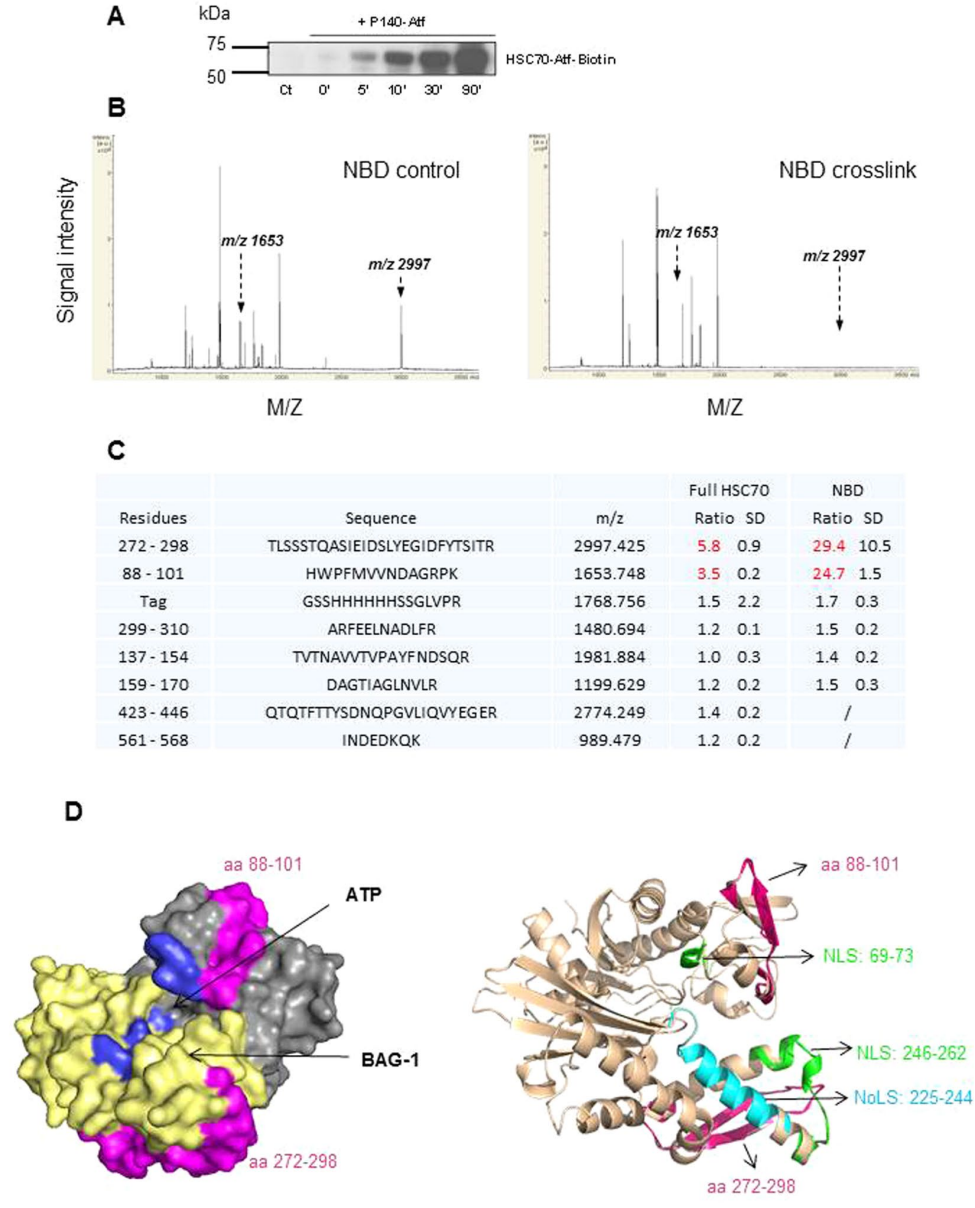
Cross-linking experiments to locate the binding site of P140 on HSPA8. (**A**) Western immunoblotting analysis showing HSPA8 protein after photo cross-linking experiments with Atf-biotin-P140 peptide (UV irradiation time is indicated in min). HSPA8 was transferred from denaturing gel to polyvinylidene difluoride membranes and stained with HRP-conjugated streptavidin, and developed with electrogenerated chemiluminescence detection reagent. (**B**) Positive reflectron MALDI-TOF spectra of HSPA8 NBD standardized as described in the materials and methods section. Trypsic digestion of NBD-containing fragment was monitored in the presence (right panel) or absence (left panel) of the cross-linking (one representative of three independent experiments with three spots/experiment). The arrows indicate the position of two peaks that vary in the cross-linked *versus* non-treated protein. (**C**) Sequence, mass and MALDI-TOF signal intensity ratios of each peptide generated in the presence or absence of cross-linking. The SD values were calculated from the 9 ratios (3 independent experiments with three spots/experiment). The ratios were considered as significant (red) if they were higher than a 3-fold change. (**D**) Left: depiction of the space-filling model of HSPA8 NBD showing the ATP pocket (blue) and the BAG-1-interacting domain (yellow). Right: Ribbon illustration of HSPA8 NBD with the amino acid sequences of interest highlighted. The location of NLS (green) and NoLS (cyan) motifs are highlighted (PDB code access 3HSC). The peptides 88–101 and 272–298, whose detection by MALDI-TOF experiments is influenced by the presence of cross-linking, are shown in fuchsia pink in both panels.

One possible mechanism to explain the striking effect of P140 on HSPA8 trafficking would be that P140 alters the NLS and/or NES of HSPA8 either by direct interaction with these signal motifs or by indirectly affecting their availability. We tested this hypothesis by using a comparative proteomics analysis of HSPA8 alone and HSPA8-P140 complex. P140 peptide labeled with a sulfhydryl-reactive methanethiosulfonate (Mts)-ultra violet (UV) light-activable perfluorophenylazide (Atf)-biotin group was led to interact with either the full-length protein or the NBD HSC70 1–386 fragment (molar ratio 1:1) and UV-irradiated. This strategy generated covalent bonds between the photoactivable group and nearby HSPA8 residues. Cross-linking efficacy was checked after purification of the resulting complex and western immunoblotting (Fig. 4A). The sequence of HSPA8 peptides involved in P140 interaction was deduced from mass spectrometry analysis of fragments produced after in-gel trypsin digestion of purified cross-linked and non-cross-linked HSPA8 protein. Standardized matrix-assisted laser desorption/ionization time-of-flight (MALDI-TOF/TOF) acquisitions revealed trypsin cleavage differences when the cross-linked protein and the unreacted one were compared. Compared to the respective non-cross-linked samples, two peptides were not significantly recovered neither in the cross-linked Atf-biotin-HSPA8 protein nor in Atf-biotin-NBD fragment (triplicate experiments and three different MALDI deposits per experiment; Fig. 4B,C; Fig. S9). These two peptides encompassed residues 272–298 and 88–101, respectively. It is anticipated that the binding of P140 peptide to HSPA8 or to the NBD, directly or at distance, hampered trypsin cleavage of the C-terminal residue of these fragments. Interestingly, depiction of the segments 88–101 and 272–298 on the models of HSPA8 and NBD-BCL2 associated athanogene 1 (BAG-1) complex (Fig. 4D) showed that they are located in the vicinity of BAG-1 and ATP binding sites identified in the crystallographic structure of complexes^10,36–38^. They are also very close to the two NLS sequences that have been identified in residues 69–73 and 246–262^5–7^ and the NoLS present in the segment 225–244^8^ of the ATPase domain (or NBD) of HSPA8. It is possible that the binding of P140 to the NBD also indirectly affects the NES encompassed in the sequence 394–401 of the substrate-binding domain^7^.

Together, these data strongly suggest that by interacting with HSPA8, P140 peptide likely interferes with the signaling motifs, which are required for its nuclear and cytoplasmic translocation in stress conditions, altering therefore its trafficking, which is a key element of cell protection in case of stress. This mechanism is clearly differently regulated according to the nature of stress.

### Does P140 interact with HSPA8 functions in the nucleus *in vivo*?

**Figure 5:**
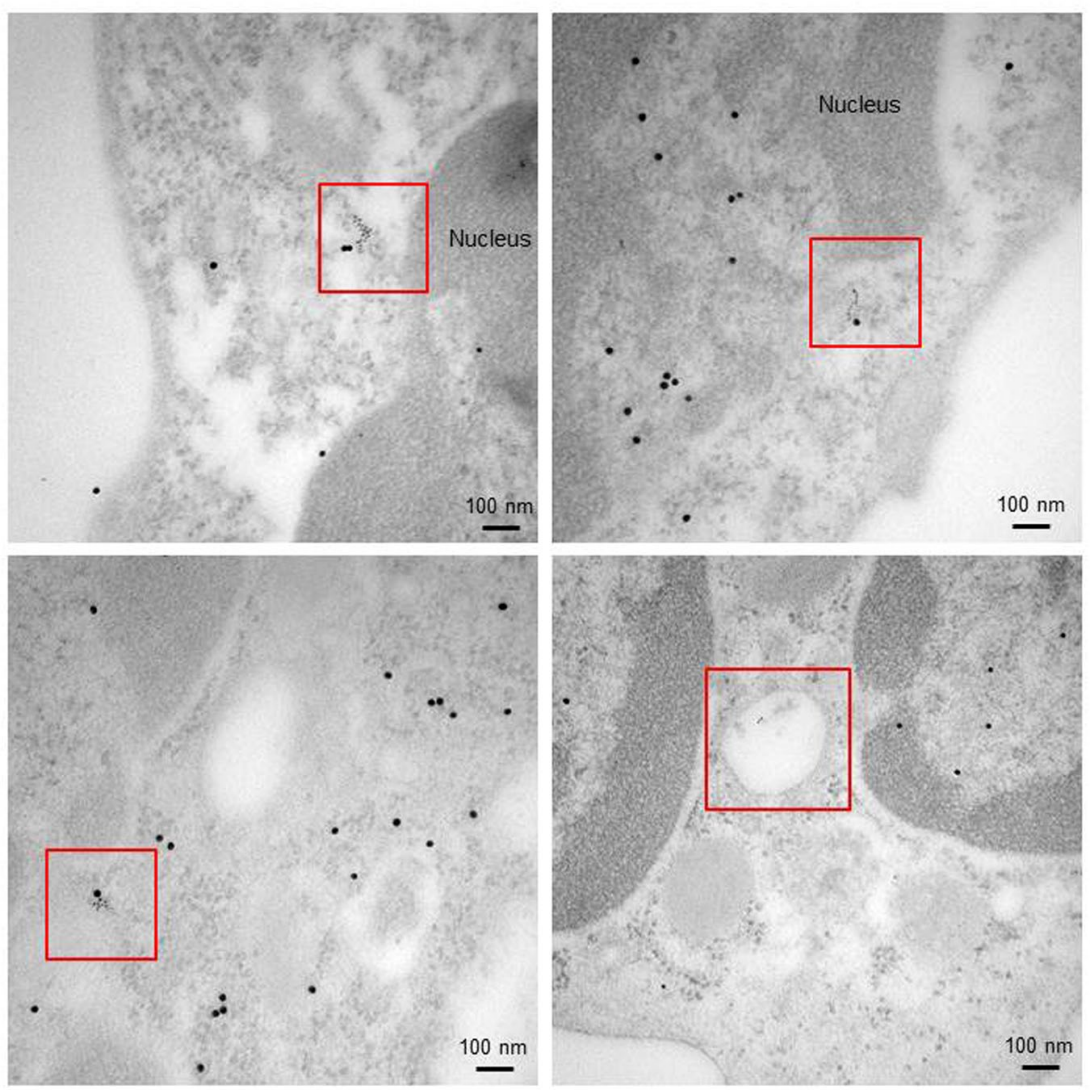
P140 peptide interacts with HSPA8 in the nucleus and hampers its functional activity. Co-localization of P140 and HSPA8 staining (red squares) as revealed by TEM in the nucleus/perinucleus (top panels) and in the cytosol (bottom left) of PBMCs collected from MRL/lpr mice that received the biotinylated P140 peptide via the intravenous route. Strings of small gold particles were also found in vesicles that might correspond to endosomes (bottom right). Immunolabeling was done using anti-biotin (for P140) and anti-HSPA8 antibodies followed by secondary antibodies. HSPA8, 15 nm-gold particles; P140, 6 nm-gold particles.

A last question remaining to solve in order to reinforce our assumption was to formally determine if P140 readily binds to HSPA8 in the nucleus of heat-shocked cells. Transmission electron microcopy (TEM) images clearly showed some dense large (HSPA8) and small (P140) gold particles that co-localized into the nucleus and perinucleus of PBMCs collected from MRL/lpr mice 1 h after intravenous injection of a biotinylated version of P140 peptide (Fig. 5; Fig. S10). These results indicate that P140 can translocate within the nucleus of cells where it can readily interact with HSPA8 and potentially block it activity as outlined above.

## Discussion

Nucleocytoplasmic shuttling of HSPA8 is crucial to protect stressed cells from possible damages and ensure their survival when the alterations that are generated are moderate. In case of stress this “in and out” trafficking that occurs at a basal level under normal growth conditions is rapidly activated by some signals and molecular mechanisms that remain largely unknown^12^. As in contrast to other Hsps, the constitutively expressed HSPA8 protein is particularly abundant in the cytoplasm, its transient egress from the cytoplasm has relatively few functional effects if any. However, its location in the nucleus/nucleoli is vital. HSPA8 possesses a binding domain for client proteins and the three domains that compose the protein interact with an army of co-chaperones, such as HSP90. When HSPA8 is going to hide in the nucleus/nucleoli of stress cells, it carries with it and protects metabolically important cell components that are required for assuring cell homeostasis and dynamics. It is why, not only the nuclear shuttling of HSPA8 but also its cytoplasmic release in the recovery phase is crucial. Numerous candidate proteins may thus been protected by the HSPA8 chaperone. These proteins can be of cytosolic origin but also, such as the nucleolar protein nucleolin^39^, localized at the cell surface.

Using the P140 peptide as a tool that binds HSPA8 and inhibits some of its functions, especially its chaperoning properties^15–17^, we report here that blocking nuclear export of HSPA8 and its client proteins in the post-HS stress recovery phase is deleterious for cell survival. Unexpectedly, the lack of normalcy restoration of HSPA8 location in the cytoplasm has no effects on cell integrity if a second HS effect is applied and does not affect the cell cycle machinery or certain key pathways of autophagy or of cell signaling such as the PI3K-Akt and Ras-Raf-MEK*-*ERK pathways. However, when an oxidative stress is subsequently applied after HS, even if a period of total recovery is allowed to occur between the two sets of shock, then cells show vulnerability and die. This series of events is remarkable since the oxidative stress we applied (submillimolar levels of H_2_O_2_) caused no effect when it was given alone without prior HS stress. Therefore, we show that the morphological and metabolic changes that likely occur when H_2_O_2_ is added to pre-sensitized heat-shocked cells dramatically affect the integrity of cells in which the HSPA8 nucleocytoplasmic repartition is not at its steady-state level. Such senescence-like morphological changes have been demonstrated previously in mouse gingival fibroblasts^40^. DNA lesions were also observed when low concentrations of H_2_O_2_ were used^41^.

Since we know that HSPA8 is a necessary cofactor for ubiquitination and degradation of a number of cellular proteins^42^, a lower availability of HSPA8 might affect the ubiquitin-mediated proteasomal degradation pathway, an important mechanism to control protein load in the cells. It could also have altered macro- and chaperone-mediated autophagy processes, an effect that was however not demonstrate in our studies. This lack of apparent consequence is probably related to the huge amount of HSPA8 that resides in the cytoplasm.

The P140 peptide tool was of instrumental value to reveal the crucial cell protective role of HSPA8 in stress conditions. A complete knock-down of HSPA8 (e.g. with siRNA) results in a massive cell death. P140, however, acts in a more subtle way. On a mechanistic point of view, we demonstrated that P140 binds to sites that may affect HSPA8 translocation, in particular in its exportation phase to the cytoplasm during the recovery phase. As we know that P140 binds to residues in the NBD^17^, it should not affect the oligomeric state of HSPA8 that involves the C-terminus of the protein^43,44^.

Accumulating studies have pointed out the potential of blocking HSP70 family members to a therapeutic way of intervention, especially in cancer, autoimmunity and neurodegeneration^1,2,14,44–46^. HSPA8 is effectively involved in MHC class II antigen presentation^21–25^ and as such, the level of its expression potentially modulates the downstream immune response. HSPA8 is also centrally implicated in highly regulated processes of cell death. To exploit the degradative outcome of stressed cells by P140 peptide might therefore constitute a valuable therapeutic strategy for precisely targeting sensitized cells and precipitate them into a cell death circuit. Alone on normal cells, P140 displays no apparent effect; its action on stressed cells, however, could be decisive to specifically eliminate distressed, sensitized cells.

## Methods

### Synthetic peptides and HSPA8

P140 peptide and scP140 analogue, as well as P140 peptide containing an additional cysteine residue at its N-terminus, biotinylated P140 and AlexaFluor 488 Cys-P140 were synthesized and purified as described^17^. Their homogeneity was checked by analytical high performance liquid chromatography (HPLC), and their identity was assessed by LC/MS on a Finnigan LCQ Advantage Max system (Thermo Fischer Scientific). Conjugation of the Mts-Atf-biotin reagent (Pierce) with SH group of the Cys-P140 peptide was performed as described by the manufacturer. After completion of the reaction, the conjugated Mts-Atf-Biotin-P140 peptide was purified by HPLC. Cloning, expression and purification of the recombinant human HSPA8 and HSPA8 NBD fragment were described previously^17^.

### Cell culture

MRL/N-1 cells were obtained from Tetsuya Kodama, Tohoku University School of Medicine, Japan. They were routinely cultured in Roswell Park Memorial Institute medium (RPMI) 1640 buffered-medium supplemented with 10% (v/v) fetal bovine serum (Dutscher), 4-(2-hydroxyethyl)-1-piperazineethanesulfonic acid (HEPES) and gentamycin (10 µg/ml; Lonza BioWhittaker). The absence of mycoplasma contamination was systematically ensured.

### Heat shock and oxidative stresses and recovery

MRL/N-1 cells were seeded for at least 6 h before further treatment. They were pre-treated or not with 10 µM P140 for 16 h, then transferred at 45 °C for 30 min (HS) and subsequently allowed to recover at 37 °C for indicated time points. P140 peptide was present all along the time, during the HS step and cell recovery. Oxidative stress was induced with 100 µM H_2_O_2_ (Sigma).

### Fluorescence imaging

After treatment, cells were fixed and permeabilized with 100% (v/v) pre-cooled methanol at −20 °C, followed by incubation with phycoerythrin (PE)-labeled antibody to HSPA8 (Abcam, ab65170) overnight at 4 °C. The nuclei were stained with 4′,6-diamidino-2-phenylindole (DAPI). Cells were then mounted with mounting medium (DAKO) and subjected to fluorescence imaging using a spinning-disc confocal microscope consisting of a CSU confocal spinning disk unit (Yokogawa), an Electron Multiplying Charge Coupled Device (EMCCD) Evolve camera (Roper Scientific), mounted on an Axio Observer Z1 microscope (Zeiss). 405 and 561 nm lasers, 420–470 nm band pass and 570–640 nm band pass emission filters were used to image the DAPI and HSPA8-PE, respectively. Quantification of fluorescence signals was done as described in Fig. S1B in using Image J software (National Institutes of Health).

### Electron microscopy

The methods used have been described in details previously^15,16^. Freshly isolated PBMCs from 9-week-old MRL/lpr mice that received 100 µg biotinylated P140 or ScP140 peptides intravenously were collected 1 h post-injection, washed and fixed in 4% (v/v) paraformaldehyde and 0.2% (v/v) glutaraldehyde in 0.1 M phosphate-buffered saline (PBS) for 1 h at room temperature (RT). Cells were embedded in 1% (w/v) agarose, permeabilized with 0.2% saponin for 10 min, washed and saturated with 2% (w/v) bovine serum albumin and 0.2% (w/v) water fish skin gelatin (Sigma) in Tris-buffered saline. HSPA8 was revealed by using anti-HSPA8 rat monoclonal antibody (1:200; clone 1B5; Abcam) and goat anti-rat IgG conjugated to 15 nm-gold particles (Aurion); P140 and ScP140 were revealed with anti-biotin antibodies conjugated to 6 nm-gold particles. Ultrathin sections were stained with uranyl acetate and lead citrate and examined by TEM using a Hitachi H600 microscope. Images were acquired using a CDD camera (Hamamatsu).

### Cell fractionation and western blot analysis

Cell fractionation, all along performed at 4 °C or on ice, was done as described^47^. Briefly, 500,000 MRL/N-1 cells were collected after treatment with trypsin, followed by one wash with cold PBS and then lyzed with lysis buffer [50 mM Tris-HCl, pH 7.0, 0.5% (v/v) Triton X-100, 137.5 mM NaCl, 10% (v/v) glycerol, 5 mM EDTA, supplemented with fresh protease inhibitors] on ice for 15 min. The nuclear pellet collected after a 5-min centrifugation at 800 *g* was washed twice in the lysis buffer and centrifuged at 800 *g*, and finally lyzed in 0.5% (v/v) sodium dodecyl sulfate (SDS)/lysis buffer (“nuclear” fractions). The supernatant was collected and centrifuged at 16,000 *g* for 10 min to obtain the clean “cytosolic” fraction. The protein concentration of both fractions was estimated with the Pierce BCA (bicinchoninic acid) protein assay (Thermo Fisher Scientific). Nuclear and cytoplasmic cellular fractions were mixed with Laemmli buffer (BioRad) and denatured for 10 min at 95 °C. The proteins were resolved by SDS-PAGE using 4–20% pre-casted gels (BioRad) and then transferred onto polyvinylidene difluoride membranes (BioRad) for immunoblot experiments. Autophagy activity was measured as described^48^ Antibodies against HSPA8 (Abcam, ab19136), HSP90 (Abcam, ab13492), histone H3- horseradish peroxidase (HRP, Cell Signaling, 5192), phospho-Akt (Cell Signaling, 9271), phospho-ERK (Cell Signaling, 9101), SQSTM1/p62 (Abcam, ab109012), LC3-II/MAP1LC3-II (MBL International Corporation, M186–3), and LAMP2A (Abcam, ab18528) were used at the indicated dilutions. HRP-conjugated secondary antibodies and an enhanced chemiluminescence method (ECL, BioRad 1705060) were used to reveal the reactions. Protein densitometry was normalized to β-Tubulin loading control (Abcam, ab21058). Protein densitometry was normalized to either the level of H3 (Cell Signaling, 12648) for nuclear fractions or that of β-Tubulin (Abcam, ab21058) for cytosolic and total fractions.

### Cell death measurement and cell counting

The fluorescent exclusion dye PI (Sigma, P4864) was used to label the nucleus in dying cell. Permeability to PI was evaluated by flow cytometer (PI^+^ cells; FACSCalibur, BD Biosciences). The cell number was counted using a LUNA-FL^TM^ automated cell counter (Logos Biosystems) following the manufacturer’s instructions.

### Cell cycle

MRL/N-1 cells were fixed in ice cold 70% (v/v) ethanol at 4 °C overnight followed by 3 washes in PBS and samples were treated with 0.1% (v/v) Triton X/PBS permeabilization buffer supplemented with 100 µg/ml RNase A (Sigma), at 37 °C for 15 min. 7-AAD (BD Pharmingen, 559925; 25 µg/mL) was added to stain the DNA, and samples were measured using a BD FACSCalibur flow cytometer (BD Biosciences) and analyzed using FlowJo software (Tree Star).

### qPCR

The mRNA of MRL/N-1 cells treated with P140 or ScP140 for indicated times and concentrations were extracted using RNeasy Mini kit (Qiagen, 75704) according to manufacturer’s instructions. The first strand cDNA was synthetized from 200 ng of RNA using ImProm-II Reverse Transcriptase and oligo(dT)_15_ (Promega, A3802 and C1101). Ten ng of cDNA were then amplified using Taqman Real-Time PCR Master Mixes (ThermoFisher Scientific, 10525395), followed by qPCR analysis of the cDNA amount using the StepOne^TM^ real-time PCR system (ThermoFisher Scientific). The primers for Hspa8 (4448892) and endogenous controls, Actb (4453320), Gusb (4453320) and Hprt1 (4453320) were purchased from ThermoFisher Scientific. The data analysis was done using the correspondent StepOne^TM^ software and the amount of Hspa8 level was normalized with the levels of the three endogenous controls.

### Cross-linking experiments

To identify the binding sites of P140 to HSPA8, 20 µL of recombinant HSPA8 or NBD (20 µM) and 20 µL of P140-Atf peptide (20 µM) corresponding to a 1:1 molar ratio of P140 peptide and HSPA8 protein/NBD fragment were incubated for 30 min at 37 °C in 100 mM Tris-HCl pH 7.4, 6 mM MgCl_2_ and 20 mM KCl. The resulting solution was then UV-irradiated at 365 nm for 20 min at 4 °C. The UV lamp (Bioblock Scientific, VL-4C) was placed at 2 cm above the sample. The complexes formed between the Atf-biotin-P140 and HSPA8 were isolated by purification of the extracts using 100 µL avidin-agarose (ImmunoPure Immobilized Avidin, Pierce) in PBS. After overnight incubation at 4 °C, the samples were washed extensively with the same buffer. The purified proteins were subjected to SDS-PAGE and revealed by colloidal blue staining. In some experiments, the efficiency of photo cross-linking was evaluated by western blotting using peroxidase-labeled streptavidin. The identification of HSPA8 peptide involved in P140 peptide interaction was deduced from mass spectrometry analysis as described below after cutting bands of interest. The in-gel digestion procedure was carried out as described^49^. Briefly, gel bands were washed alternately with 100 µL of 25 mM NH_4_HCO_3_ and then 100 µL of 50% (v/v) acetonitrile (ACN) (3 min wash under shaking and the liquid was discarded before addition of the next solvent). This hydrating/dehydrating cycle was repeated twice and the pieces of gel were dried for 20 min before reduction (10 mM dithiothreitol/25 mM NH_4_HCO_3_ for 45 min at 56 °C) and alkylation (25 mM iodoacetamide/25 mM NH_4_HCO_3_ for 45 min at RT). Gel spots were washed again with 3 cycles of 25 mM NH_4_HCO_3_/ACN, alternately. Following a 20 min-drying step, the gel pieces were rehydrated by three volumes of trypsin (Promega, V5111), 12.5 ng/µL freshly diluted in 25 mM NH_4_HCO_3_ buffer and incubated overnight at RT. Tryptic peptides were extracted from gel by vigorous shaking for 30 min in adapted volume of 35% H_2_O/60% ACN/5% HCOOH and a 15-min sonication step. MALDI mass measurements were carried out on an Autoflex III Smartbeam MALDI-TOF/TOF spectrometer (Bruker-Daltonik) used in positive mode, over a mass range of 700–4,000 Da (reflector mode) and 700–8000 Da (linear mode). A pre-spotted anchor chip target (PAC system, Bruker-Daltonik, technical note TN011) with HCCA matrix was used to analyse tryptic digests. In order to compare MALDI spectra for the whole set of samples, laser intensity was set to the same value for all of them (no deflection from the baseline and average resolution of 8,000 over the whole mass range), as well as the number of shots (sum of 2,000 shots by group of 500 shots). Moreover, three different MALDI deposits and mass measurements were made for each sample and a ratio was determined according to signals intensities for each condition (cross-linked protein and non-cross-linked protein).

### Structure depiction

The spacing filling and ribbon model of HSPA8 NBD (PDB: 3HSC) was depicted using PyMOL software.

### Statistical analyses

Statistical analyses were performed using GraphPad Prism version 5.0. Depending on the number of samples that were included in the analyses, statistical significances were assessed using the parametric Student’s *t*-test or the non-parametric Mann-Whitney’s test when one parameter was included. Two-way ANOVA was used when two parameters were compared. Depending on the number of samples, non-parametric or parametric were used. *P* values less than 0.05 were considered significant. In crosslinking experiments, the standard deviation (SD) values were calculated from the 9 ratios (3 independent experiments with three spots per experiment). The ratios were considered as significant if they were higher than a 3-fold change.

### Ethics Statement

Animal protocols were carried out with the approval of the local Institutional Animal Care and Use Committee (CREMEAS, Strasbourg, France) and the French Ministère de l’Enseignement Supérieur de la recherche et de l’innovation (procédure APAFiS, autorisation de projet utilisant des animaux à des fins scientifiques). According to our agreement, and taking into account the best European practices in the field, we took the necessary measures to avoid pain and minimize the distress and useless suffering of mice during the time of experiment and killing process.

## Electronic supplementary material

Supplementary Dataset 1
