# Supplementary material for: Blocking nuclear export of HSPA8 after heat shock stress severely alters cell survival
Source: Sci Rep. 2018 Nov 14;8:16820. doi: 10.1038/s41598-018-34887-6 (PMC6235846; doi:10.1038/s41598-018-34887-6)
Supplement: Supplementary file 1 — Supplementary Dataset 1 [file 41598_2018_34887_MOESM1_ESM.pdf]

Blocking nuclear export of HSPA8 after heat shock stress severely alters cell survival

Fengjuan Wang (a), Srinivasa Reddy Bonam (a) , Nicolas Schall (a), Lauriane Kuhn (b), Philippe Hammann (b), Olivier Chaloin (c), Jean-Baptiste Madinier (a), Jean-Paul Briand (a), Nicolas Page (d) and Sylviane Muller (a,e)

(a) CNRS-University of Strasbourg, Biotechnology and cell signaling, Illkirch, France/Laboratory of excellence Medalis, France;
(b) CNRS, Proteomics Facilities, Institut de Biologie Moléculaire et Cellulaire, Strasbourg, France;
(c) CNRS, Immunology, Immunopathology and Therapeutic Chemistry, Institut de Biologie Moléculaire et Cellulaire, Strasbourg, France;
(d) Department of Pathology and Immunology, CMU-University of Geneva, Geneva, Switzerland;
(e) University of Strasbourg Institute for Advanced Study, Strasbourg, France.

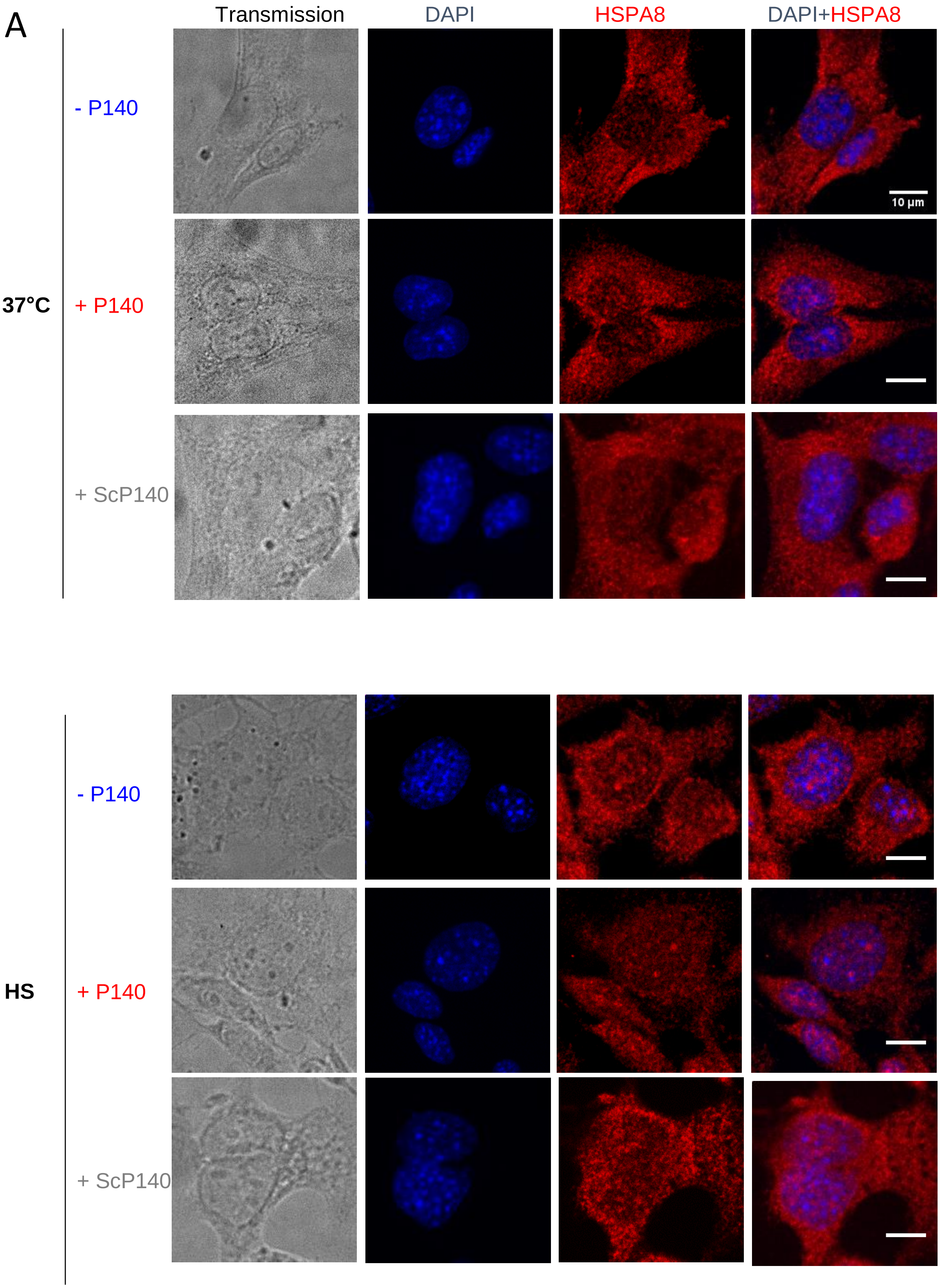
A
Transmission
DAPI
HSPA8
DAPI+HSPA8
37°C
- P140
+ P140
+ ScP140
10 µm
HS
- P140
+ P140
+ ScP140

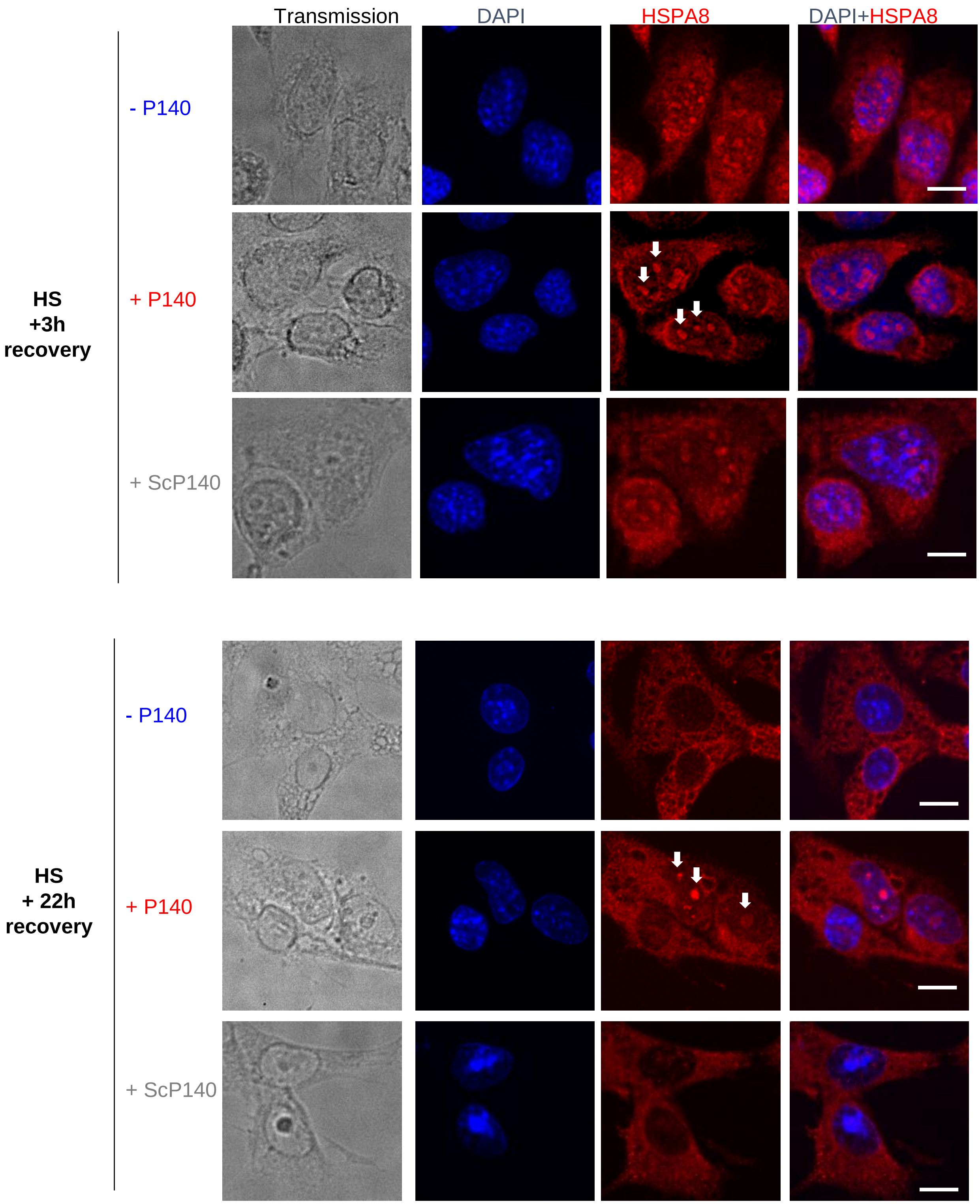
Transmission
DAPI
HSPA8
DAPI+HSPA8
HS
+3h
recovery
- P140
+ P140
+ ScP140
HS
+ 22h
recovery
- P140
+ P140
+ ScP140

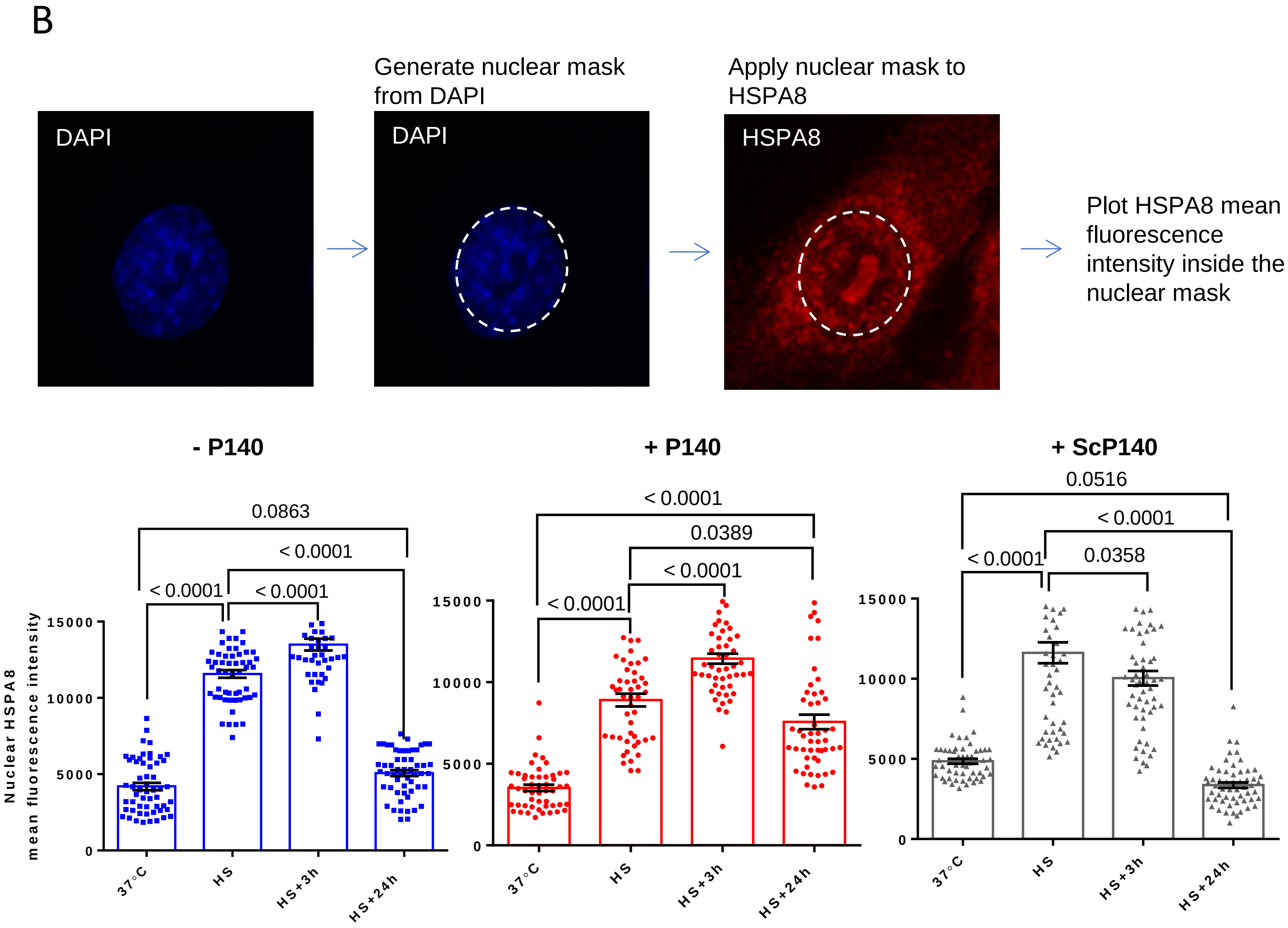

Figure S1: Confocal images of HSPA8 distribution in MRL/N-1 cells after heat shock and recovery, in the presence or not of P140 or with ScP140 control peptide. Cells were treated as described in Figure 1. **(A)** Transmission light microscopy images showing the cell morphology (grey); fluorescent confocal microscopy showing HSPA8 (red), DAPI (blue) and the overlap between HSPA8 and DAPI. The white arrows point HSPA8 that is concentrated in nucleoli that are negative for DAPI staining. (B) Quantification of the mean fluorescent intensity. A mask was created according to DAPI fluorescence channel, and was applied to HSPA8 fluorescence channel (illustrated in the top panel). The mean fluorescence intensities of HSPA8 in the DAPI mask were measured and plotted (bottom panel). Around 50 cells were analyzed. Error bars are SEM. Ordinary one-way ANOVA was used to analyse the significance of the results.

A

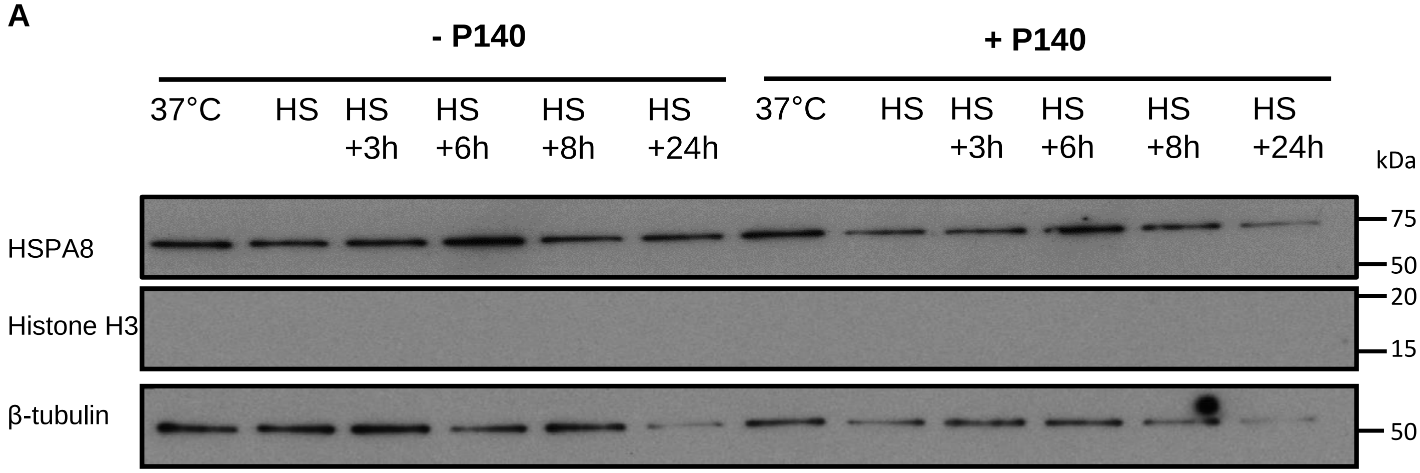

B

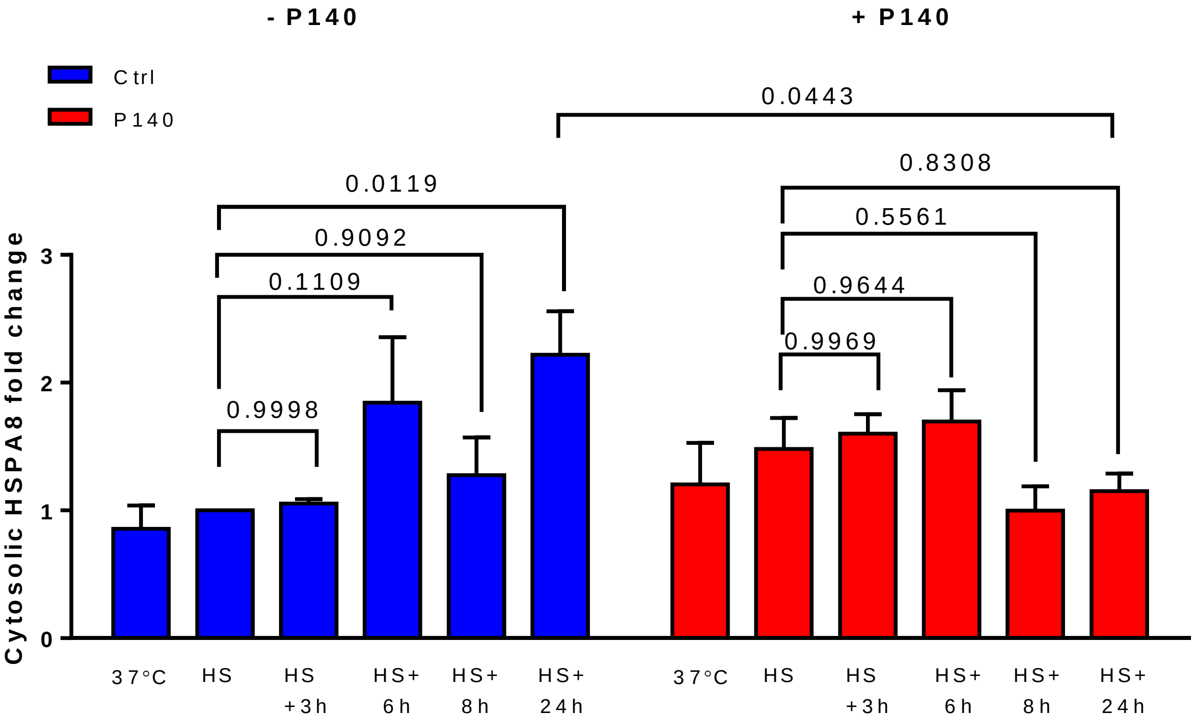

Figure S2: Western blot analysis of HSPA8 in cytosolic fractions. (A) Cells were treated as in Figure 1. The absence of histone H3 labelling indicates that the cytoplasmic fraction is not contaminated by nuclear components. β-Tubulin was used as cytosolic marker and internal protein calibrator. (**B**) The analysis was done as in Figure 1B. Error bars are SEM from 3 independent experiments (n=6). Non parametric two-way ANOVA was used to analyze the statistics.

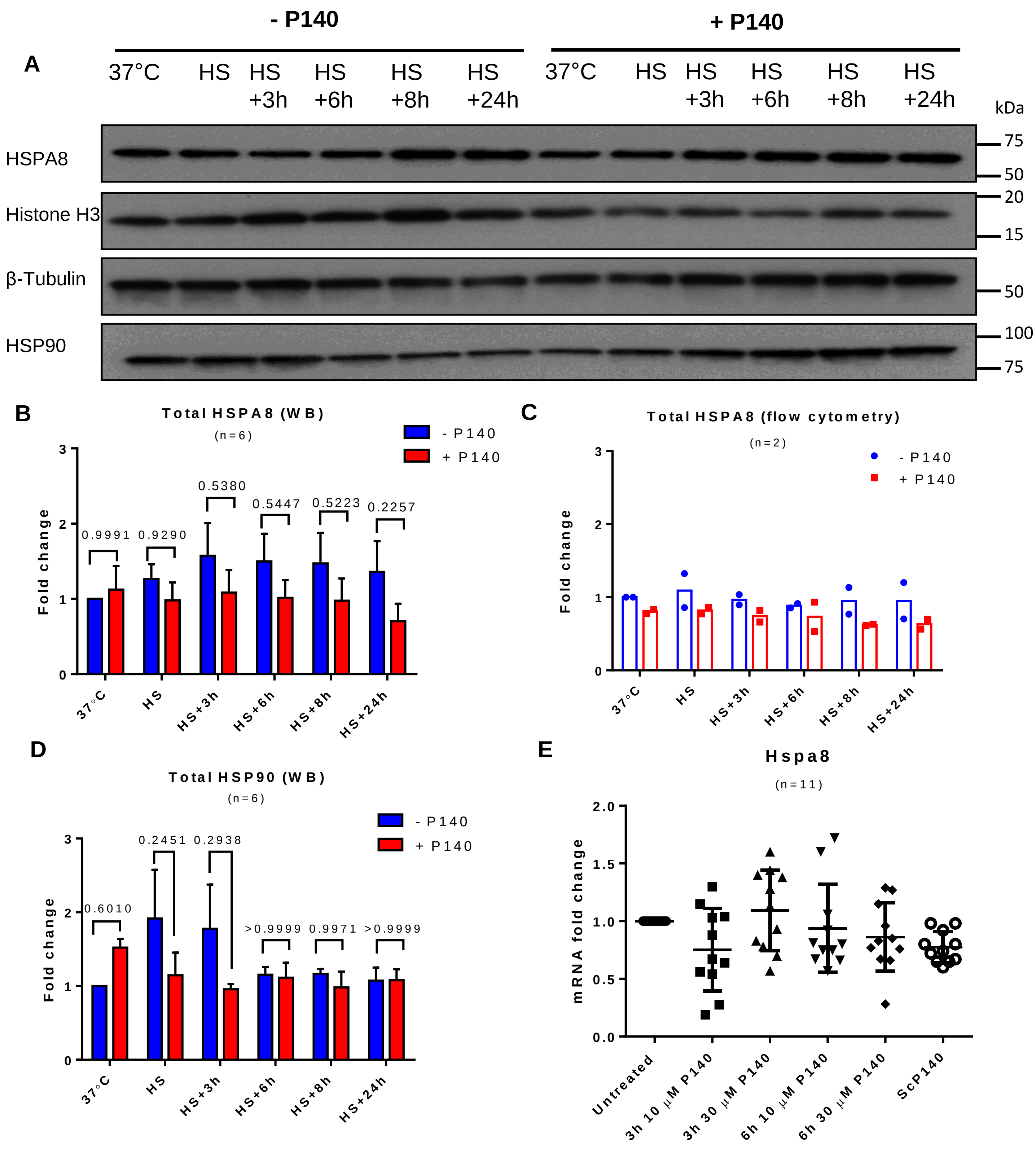

Figure S3: P140 does not affect the total protein amount of HSPA8 and HSP90. (**A**) MRL/N-1 cells were treated as in Figure 1B. The amount of HSPA8 and HSP90 from the total lysate was examined by western blot (WB). (**B**) The densitometry analysis of total HSPA8 amount was carried out by Image J as described in Figure 1B, except that all values were normalized with that of control (Ctrl) at 37°C (instead of Ctrl after HS). (**C**) Cells were treated as in Figure 1B. They were subsequently stained with HSPA8-PE antibody, followed by flow cytometry studies of total cellular HSPA8 fluorescence. The values were normalized by the mean fluorescence intensity of cells at 37°C, and the fold change was plotted. (**D**) The amount of HSP90 level in (A) was analysed using densitometry as described in (B). (**E**) MRL/N-1 cells were treated with 10 or 30 μM P140 or 30 μM scP140 for indicated times points (3 and 6h). The expression levels of Hspa8 were normalized to those of Actb, Gusb and Hprt1 used as housekeeping genes. Non parametric two-way ANOVA was used to analyse the statistics for (B-D). One-way ANOVA was used for (E) and the data do not have significance.

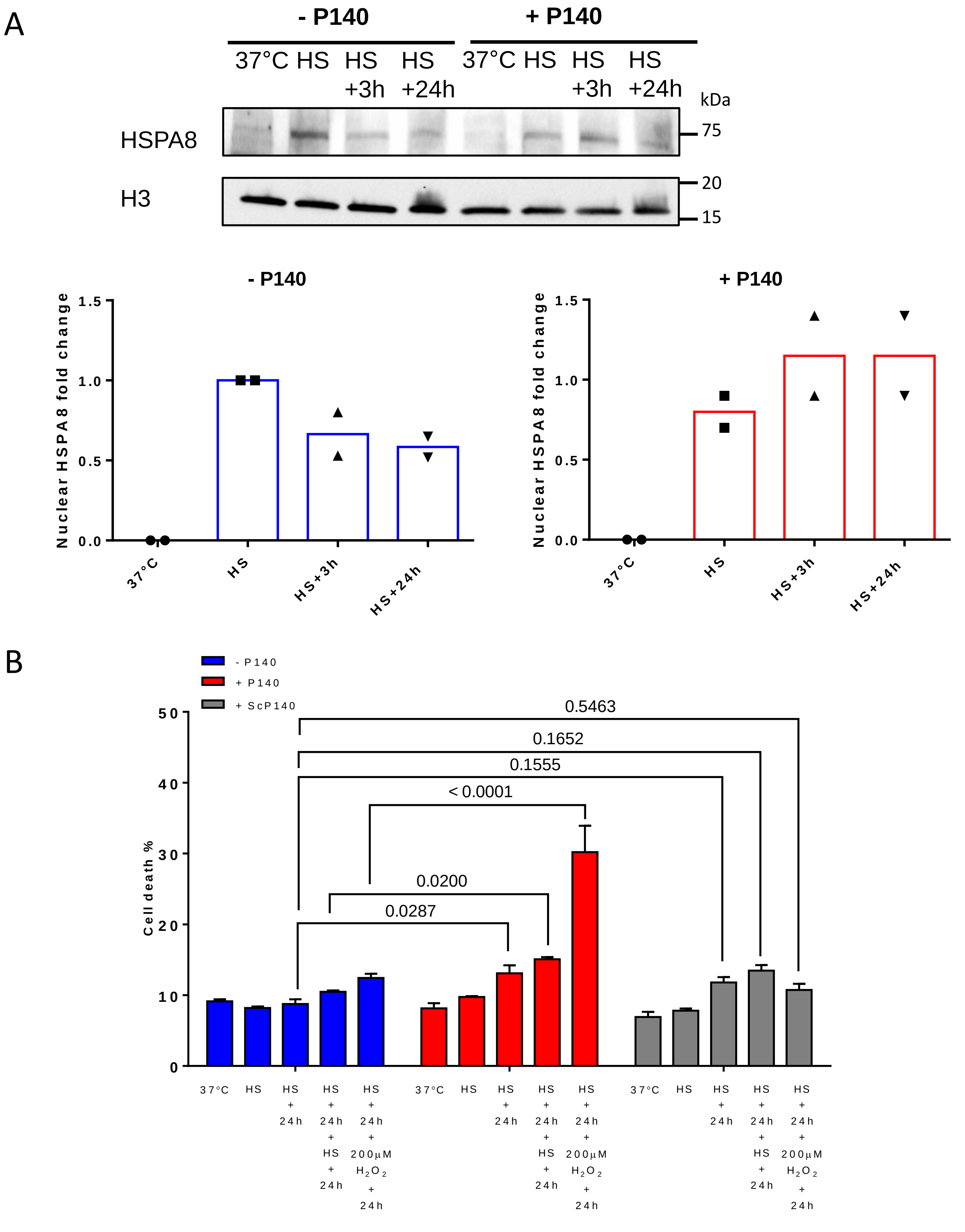

Figure S4: Inhibition effect of P140 on HSPA8 nuclear accumulation in MEF cells. (A) Western immunoblot of nuclear HSPA8 of MEF cells studied before and after HS. The experiments were performed as described in Figure 1B for MRL/N-1 cells. Histone H3 was used as internal protein calibrator. **(B)** MEFs were treated as described in Figure 3 (experiments performed as indicated in the legend). Error bars are SEM (n=4). Non parametric two-way ANOVA was used to analyze the significance of data.

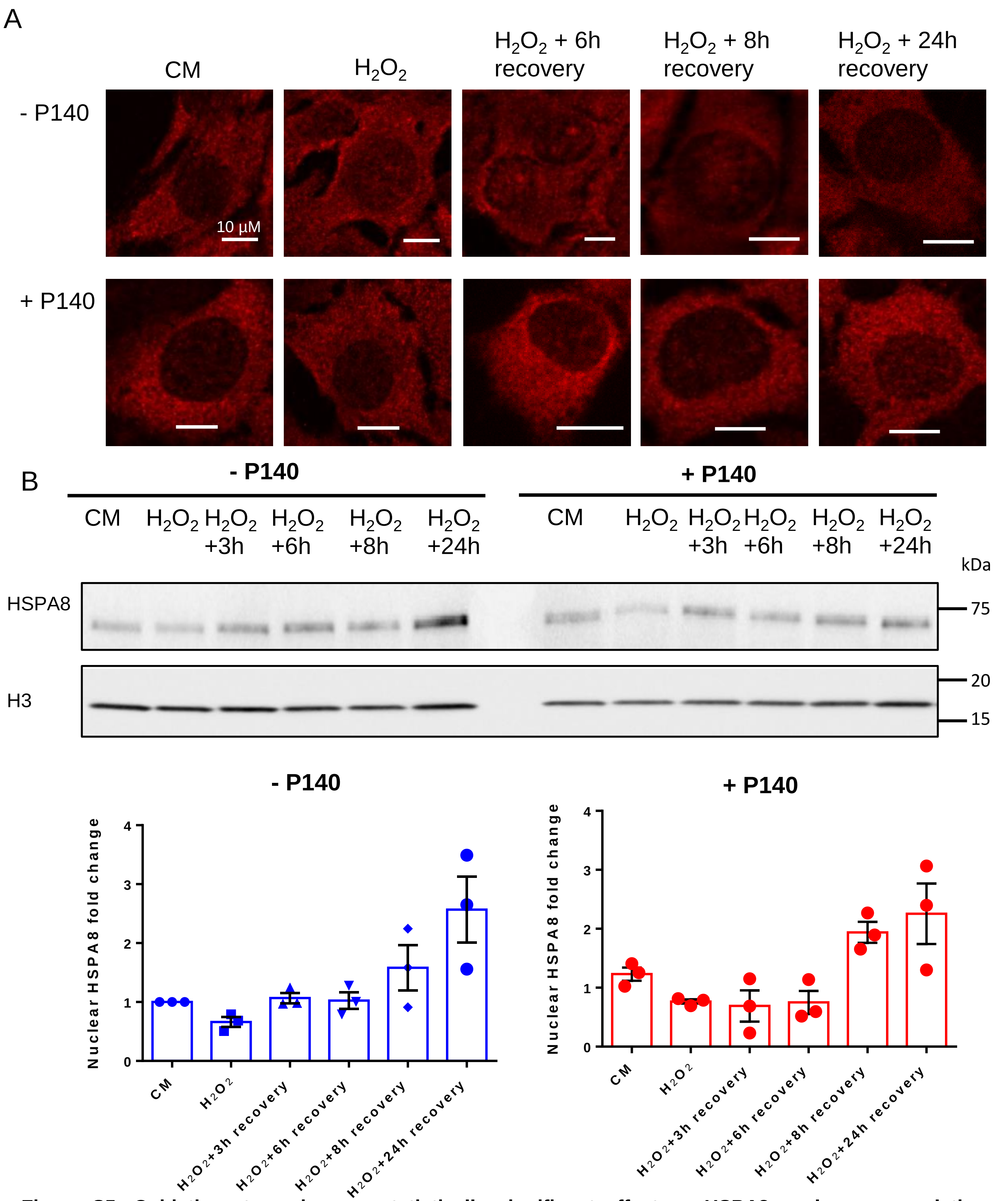

Figure S5: Oxidative stress has no statistically significant effect on HSPA8 nuclear accumulation compared to heat shock. MRL/N-1 cells were exposed to 100 µM H_2O_2 and allowed to recovery in fresh complete medium (CM) for indicated time points, in the absence or presence of 10 µM P140. Cells were either subjected to confocal fluorescence imaging (**A**) as described in Figure 1A, or to cell fractionation to examine the nuclear HSPA8 amount by western blot (**B**) as in Figure 1B. Histone H3 was used as internal protein calibrator. Error bars are SEM (n=3).

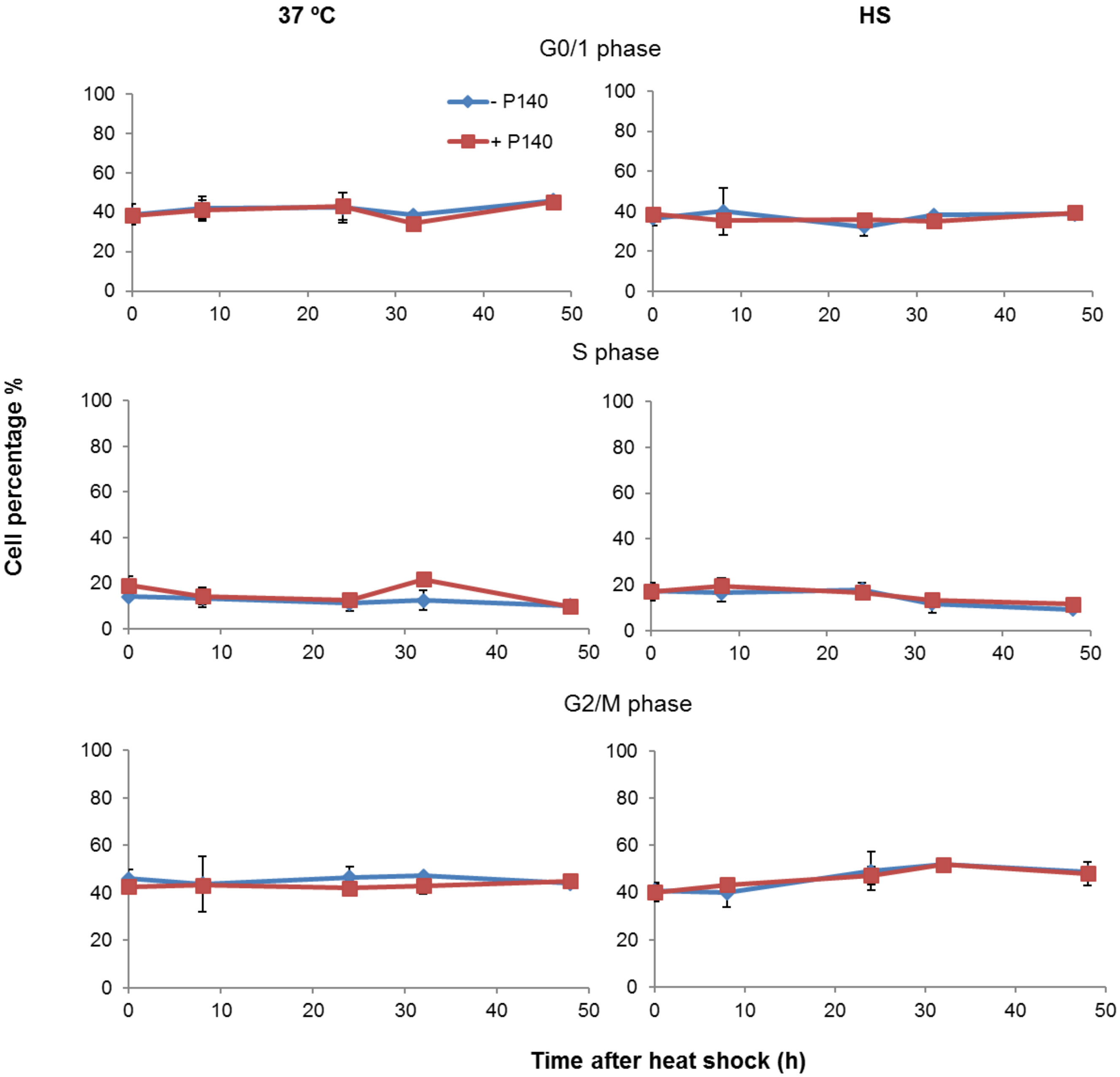

Figure S6: P140 does not affect the cell cycle after heat shock. MRL/N-1 cells were treated as in Fig. 2. The cell cycle phases were analysed using flow cytometry after PI staining of DNA. The results are from 2 independent experiments with 2 replicates each time. Error bars are SEM (n=4).

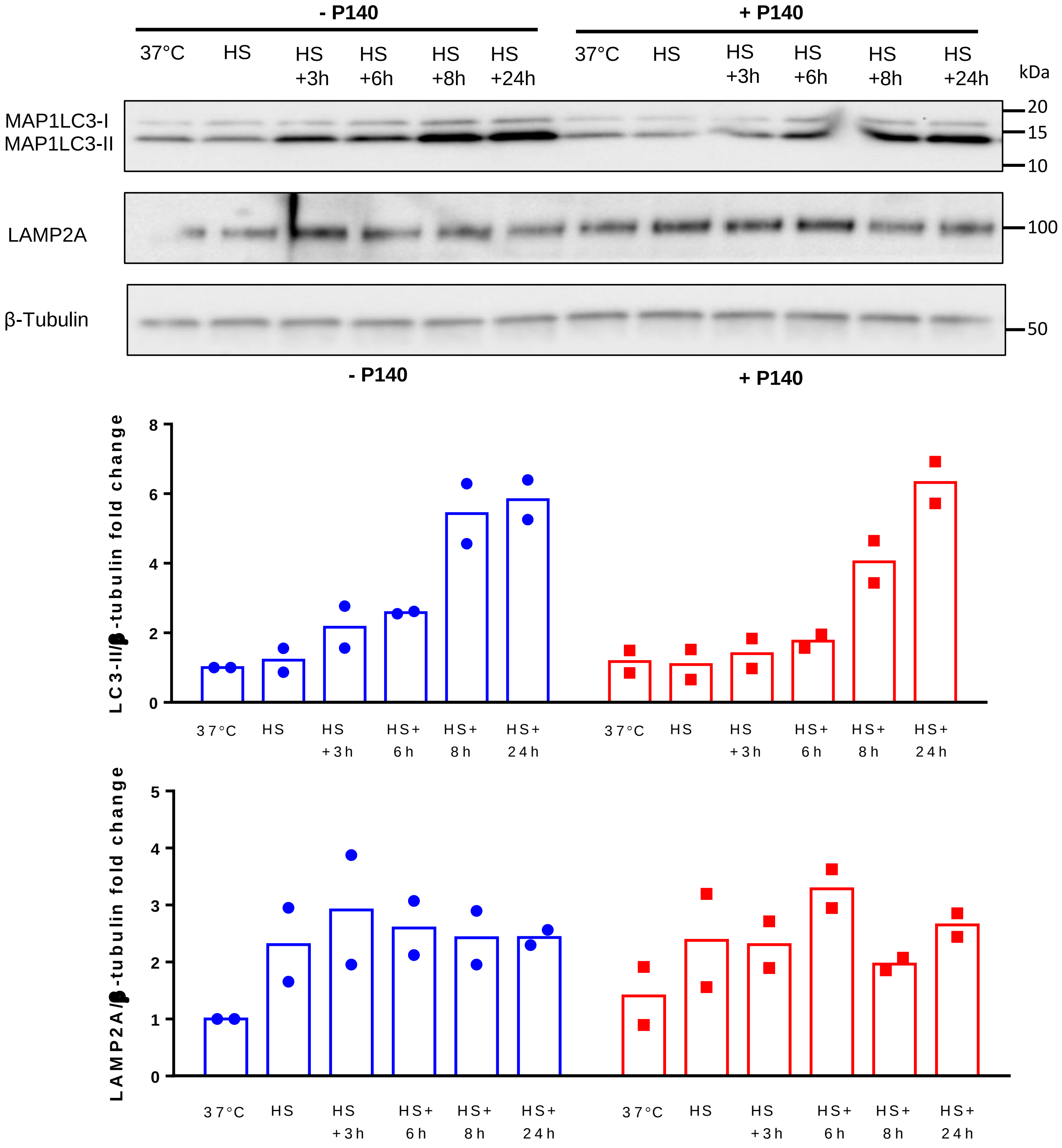

Figure S7: P140 does not affect macroautophagy and CMA. MRL/N-1 cells were exposed to HS and allowed to recover at 37°C for the indicated times in the presence or absence of P140 as in Fig. 1B. Cell lysates were subjected to western blot analysis of MAP1LC3-I/II and LAMP2A, which are indicators of macroautophagy and CMA, respectively. β-Tubulin was used as internal protein calibrator. Two individual experiments were performed.

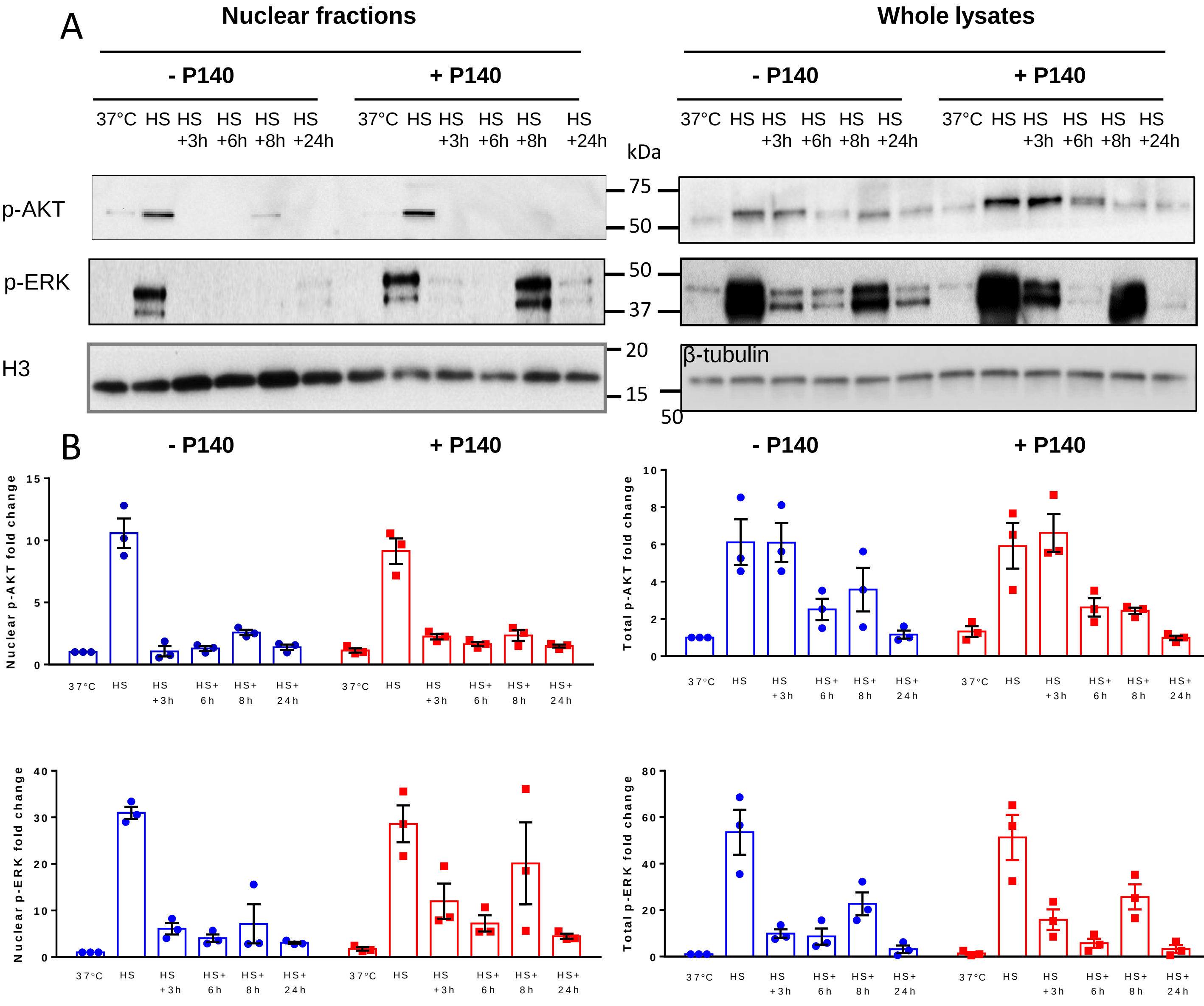

Figure S8: P140 does not affect PI3K-AKT nor MEK-ERK1/2 pathway. MRL/N-1 cells were exposed to HS and allowed to recover at 37 °C for indicated times in the presence or absence of P140 as in Fig. 1B. Nuclear fractions and whole lysates were prepared and subjected to western blot experiments to analyse the expression of p-AKT and p-ERK used here as markers of PI3K-AKT and MEK-ERK1/2 pathways, respectively. Histone H3 and β-Tubulin were used as internal protein calibrators for nuclear and whole lysate fractions, respectively. Representative blots are shown in (A) and the densitometry analyses are shown in (**B**). Three individual experiments were performed and error bars are SEM.

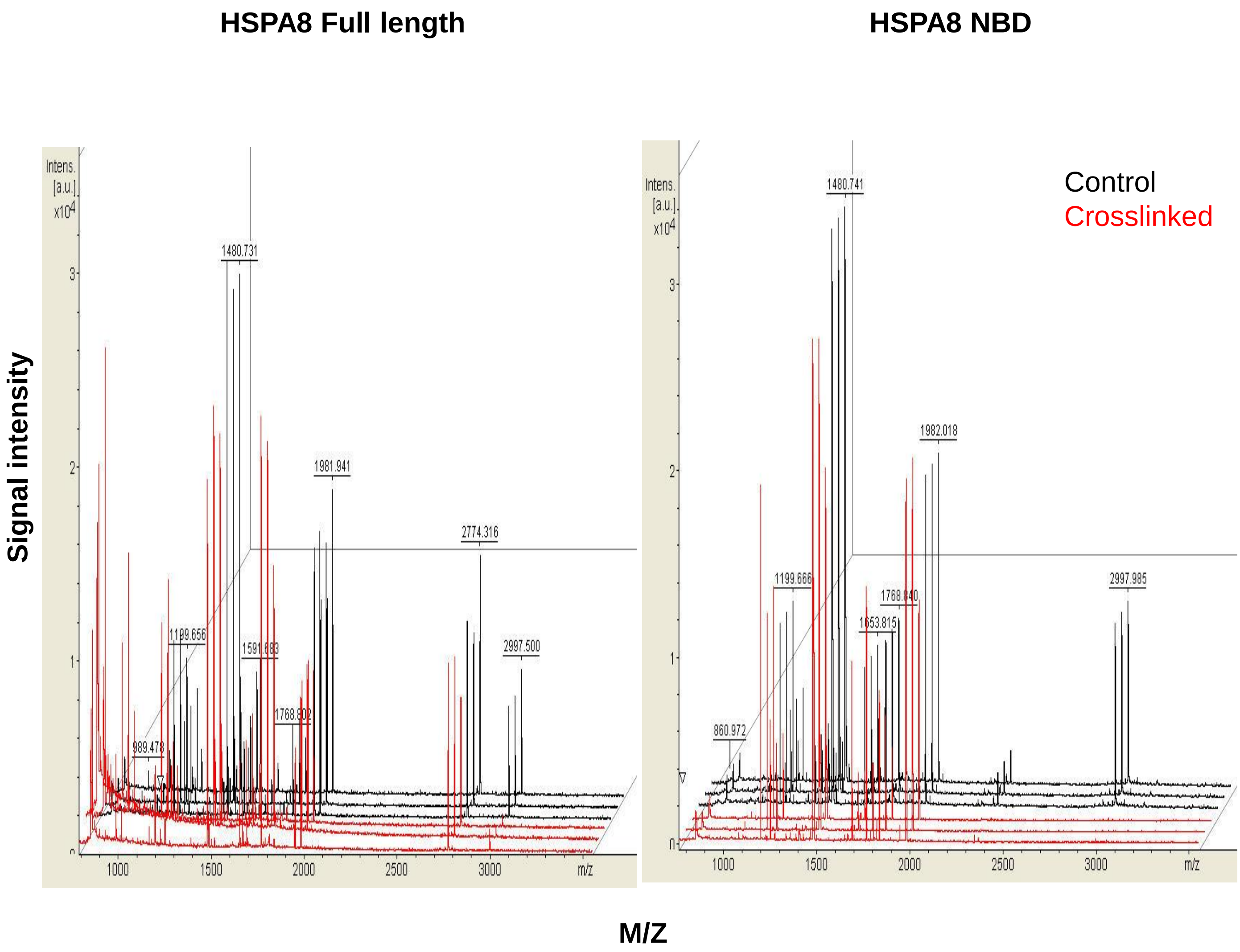

Figure S9: Mass spectrometry analysis of cross-linking experiments designed to locate the binding site of P140 on HSPA8. Tryptic in-gel digestion peptides from the whole HSPA8 protein and the NBD fragment were monitored in the presence or absence of the photo cross-linking (three independent experiments with three spots/experiments). The positive reflectron MALDI-TOF spectra standardized as described in the materials and methods section are shown.

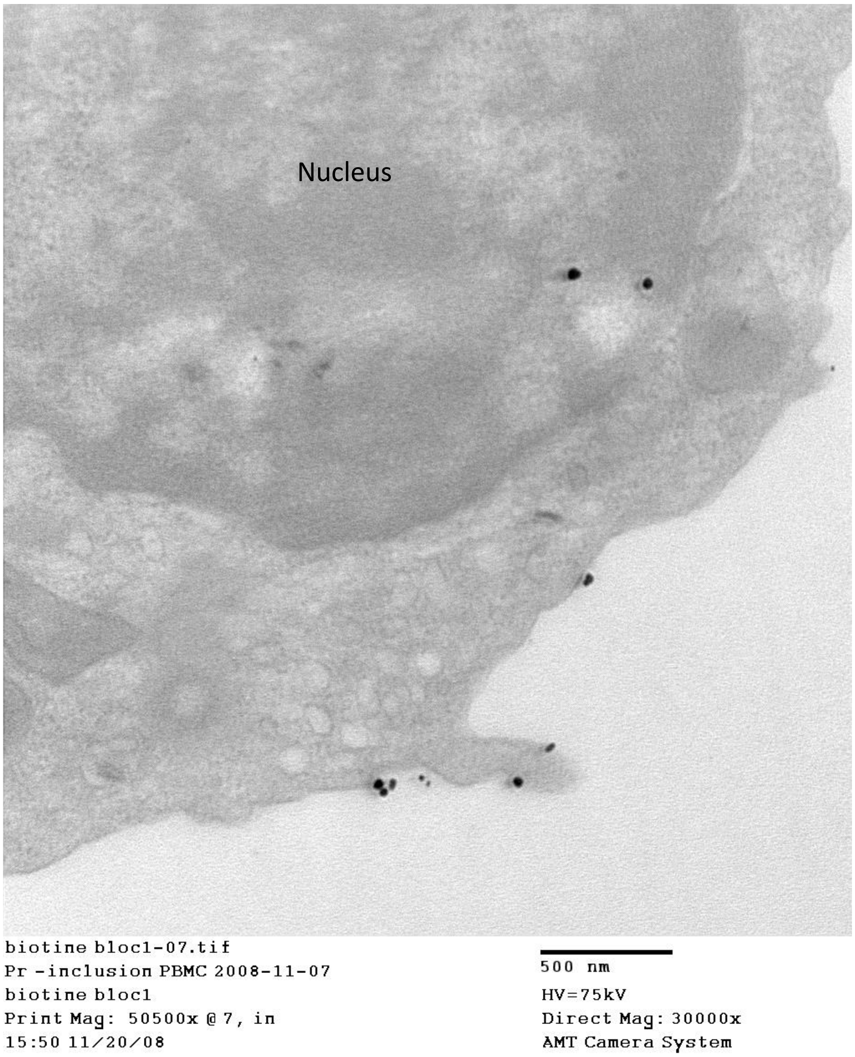

Figure S10: TEM images of PBMCs collected from MRL/lpr mice that received the biotinylated P140 peptide. MRL/lpr mice were injected intravenously with 100µg P140. PBMCs were collected 30 minutes later and prepared for TEM examination. Immunolabelling was done using anti-biotin antibody followed by secondary antibody conjugated to 6 nm-gold particles. The image shows that P140 is present on the cell membrane and within the nucleus.
